# Study on Berberine/Glycyrrhizic Acid Monoammonium Salt Self-Assembled Hydrogel for Diabetic Wound Healing

**DOI:** 10.3390/gels12090799

**Published:** 2026-09-02

**Authors:** Li Jia, Ailin Zhang, Jiayu Wang, Yingying Shen, Jianchang Huang, Weinan Li

**Affiliations:** 1 Department of Pharmacy, Heze Medical College, Heze 274000, China; 2 School of Pharmacy, Heilongjiang University of Chinese Medicine, Harbin 150040, China; 3 Key Laboratory of Basic and Application Research of Beiyao, Heilongjiang University of Chinese Medicine, Ministry of Education, Harbin 150040, China

**Keywords:** berberine, glycyrrhizic acid monoammonium salt, self-assembled hydrogel, diabetic wound healing

## Abstract

Diabetic chronic wounds face multiple intractable healing obstacles including sustained inflammation, severe infection, insufficient angiogenesis and defective collagen deposition. Current dressings fail to simultaneously relieve all these pathological defects. Herein, we constructed a carrier-free binary self-assembled Glycyrrhizic acid monoammonium salt–Berberine (GB) hydrogel composed of berberine and glycyrrhizic acid monoammonium salt, which forms interconnected nanofiber networks via one-pot thermally assisted small-molecule co-assembly without chemical crosslinking or exogenous polymer carriers. Relying on intermolecular non-covalent interactions, this single supramolecular material integrates anti-inflammatory, broad-spectrum antibacterial, pro-angiogenic, and collagen-regenerative multifunctions, which simultaneously ameliorates multiple core pathological obstacles of diabetic wounds within one formulation. We systematically characterized its physicochemical features, biocompatibility, and antibacterial and anti-inflammatory activities, as well as in vivo wound repair performance. This hydrogel formed uniform nanofibrous architectures with favorable viscoelastic properties and pH-dependent sustained release. In vitro assays verified its outstanding biosafety, broad-spectrum bacteriostasis against *Escherichia coli* and *Staphylococcus aureus*, and potent inhibitory effects on pro-inflammatory cytokines TNF-α and IL-6, with bacterial inhibition rates reaching ~88% against *E. coli* and ~70% against *S. aureus*. In diabetic mouse full-thickness infected wound models, the GB hydrogel simultaneously alleviated local inflammation, accelerated wound closure and facilitated ordered collagen deposition and mature microvessel formation, achieving a 73.10% wound closure rate at day 7 and 58.01% collagen deposition fraction at day 14, thus exhibiting equivalent or superior repair capacity compared with commercial hydrogel dressings. This carrier-free supramolecular system based on natural herbal small molecules provides a safe, convenient, and integrated therapeutic strategy for diabetic infected chronic wounds, with promising clinical translation potential.

## 1. Introduction

Diabetes is a metabolic disease characterized by chronic hyperglycemia, with a continuously rising prevalence worldwide. One of the most frequent and grave complications associated with diabetes is chronic skin lesions in diabetic patients, diabetic foot ulcers in particular. Due to the combined effects of a long-term hyperglycemic microenvironment, persistent inflammation, impaired angiogenesis, and bacterial infection, diabetic wounds often heal slowly or even fail to heal, significantly deteriorating the living standard of affected patients and putting substantial pressure on medical service systems [1,2]. Wound healing is a sophisticated physiological process involving multiple events [3,4]. For complex diabetic wounds, current clinical treatments mainly include debridement, anti-infection, and wound dressing coverage, but these suffer from long treatment cycles, poor infection control, and limited tissue regeneration efficacy. Therefore, developing novel wound dressings integrating anti-inflammatory, antibacterial, and tissue-regenerative functions is of great clinical significance.

Over the past few years, self-assembling hydrogels fabricated from natural bioactive components have garnered widespread interest in cutaneous wound repair due to their carrier-free nature, superior biocompatibility, and simple synthetic procedures [5,6,7]. Unlike traditional hydrogels that require loading exogenous active substances (e.g., nanosilver, antimicrobial peptides, insulin) to achieve antibacterial and pro-healing functions [8,9], such natural-component self-assembled systems can neutralize excessive reactive oxygen species (ROS) and regulate the local pH microenvironment through the synergistic effect supplied by their own structures and components, effectively breaking the vicious cycle of local chronic inflammation [10,11]. In view of the superior application potential of natural small-molecule self-assembled systems in chronic wound therapy, screening appropriate bioactive building blocks is critical to achieve comprehensive antibacterial, antioxidant, and pro-regenerative performance.

Considering the pathological characteristics of chronic wounds, including persistent inflammation, oxidative stress, and high infection risk, berberine (BBR) and glycyrrhizic acid monoammonium salt (GA) were rationally selected as dual functional assembly units in this work. Numerous existing studies have reported GA single-component supramolecular self-assembled hydrogels for skin repair, yet such single GA systems exhibit weak antibacterial activity against Gram-negative bacteria and lack synergistic immunomodulatory capacity to reverse the sustained inflammatory microenvironment of diabetic wounds [12]. Meanwhile, most reported berberine-based wound dressings rely on exogenous polymer carriers for drug loading, which introduces potential material-related biocompatibility risks and leads to burst drug release without sustained responsive delivery behavior [13]. BBR, an isoquinoline alkaloid derived from *Coptis chinensis* and other traditional Chinese herbs, displays broad-spectrum antibacterial, anti-inflammatory, and immunomodulatory pharmacological activities [14,15]. It can enhance the mechanical strength of nascent tissues [16] and stimulate the proliferation, migration, and tubular structure formation of vascular endothelial cells [17]. GA, the main active ingredient of licorice, possesses excellent anti-inflammatory, antibacterial, antioxidant, and self-assembly properties [18,19]. This work has been inspired by previous research showing that carrier-free binary small-molecule hydrogels constructed via electrostatic attraction, π–π stacking, and hydrogen bonds can achieve combined antibacterial and wound-healing effects [20]. Numerous investigations have verified that GA is capable of self-assembling into nanofiber networks within water solutions through noncovalent forces including hydrogen bonding interactions [10,21], enabling the encapsulation of hydrophobic drugs and improving their solubility [22]. Benefiting from the structural complementarity and functional synergy of BBR and GA, the carrier-free binary co-assembly strategy proposed in this work avoids additional polymer crosslinkers and exogenous drug carriers. Compared with previously reported single GA hydrogels and carrier-loaded BBR dressings, the Glycyrrhizic acid monoammonium salt–Berberine (GB) hydrogel system integrates complementary broad-spectrum antibacterial properties, synergistic anti-inflammatory activity, pH-triggered sustained release, and carrier-free biosafety advantages simultaneously, effectively overcoming the core drawbacks of existing similar materials. This work further enriches the construction logic of herbal small-molecule supramolecular biomaterials and provides a feasible experimental basis for the development of integrated diabetic wound dressings.

**Scheme 1:**
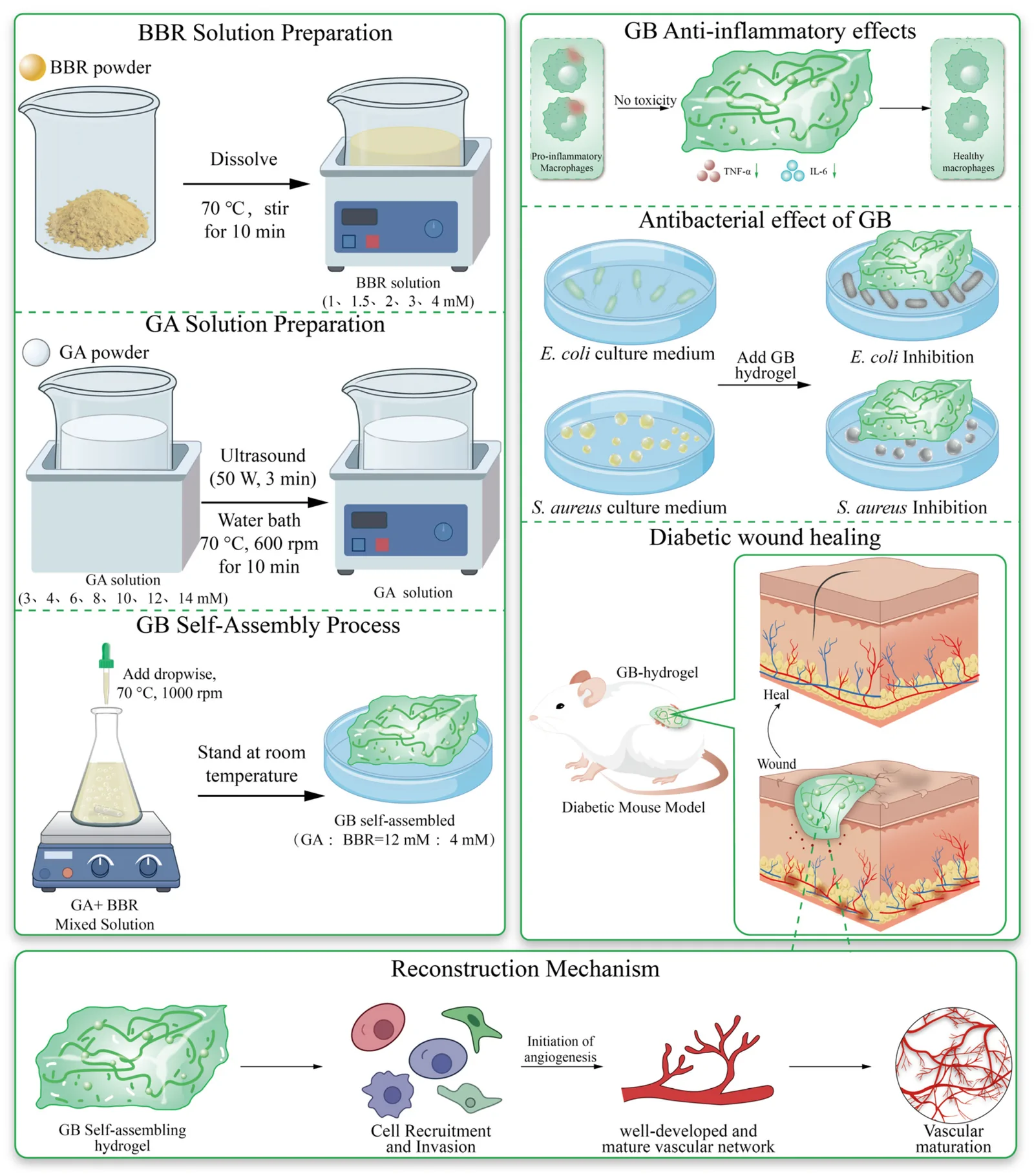
Schematic illustration of the fabrication, biological activities, and therapeutic mechanism of GB self-assembled hydrogel for diabetic wound healing. The fabrication process involves the preparation of BBR and GA solutions, followed by a self-assembly procedure to form the GB hydrogel. The as-prepared GB hydrogel exhibits favorable anti-inflammatory effects (with no toxic effects exerted on macrophages, while inhibiting the pro-inflammatory cytokines tumor necrosis factor-α (TNF-α) and interleukin-6 (IL-6) and antibacterial activity against *Escherichia coli* and *Staphylococcus aureus.* In a diabetic mouse model, the GB hydrogel promotes wound healing by recruiting endogenous cells, initiating angiogenesis, and facilitating the formation of a mature vascular network to accelerate tissue regeneration.

Herein, we developed a carrier-free, simple-component, and biosafe supramolecular hydrogel using BBR and GA as building blocks, which co-assembled into a stable self-assembled hydrogel system (GB) via intermolecular non-covalent interactions (hydrogen bonds, π–π stacking, and electrostatic interactions). Without chemical cross-linking or additional carriers, GB integrates the inherent pharmacological activities and self-assembly characteristics of the two agents to achieve combined antibacterial and anti-inflammatory effects, offering a safe, effective, and non-invasive topical therapeutic strategy for diabetic chronic wounds. Critically, it may supply a new idea and experimental support for the development of functional dressings based on self-assembly of traditional Chinese medicine active ingredients (Scheme 1).

## 2. Results and Discussions

### 2.1. Results

#### 2.1.1. Optimization of Preparation Process

##### Macroscopic Characterization

GA alone could spontaneously form a colorless transparent gel at 12 mM in the aqueous phase. After the introduction of BBR, the system turned into a uniform yellow color, preliminarily indicating the uniform distribution of BBR within the GA nanofiber matrix. Further formulation screening showed that GA concentration played a dominant role in gelation. When GA concentration reached 12 mM and above, intermolecular non-covalent interactions were significantly enhanced, enabling the formation of a denser three-dimensional network with certain mechanical strength. With GA concentration fixed at 12 mM, the effect of BBR ratio was investigated. Results showed that BBR did not disrupt the gel network; combined with subsequent FT-IR, XRD, ^1^H-NMR and rheological characterizations, BBR participated in intermolecular non-covalent interactions and exhibited an assisting co-assembly behavior with GA.

**Figure 1:**
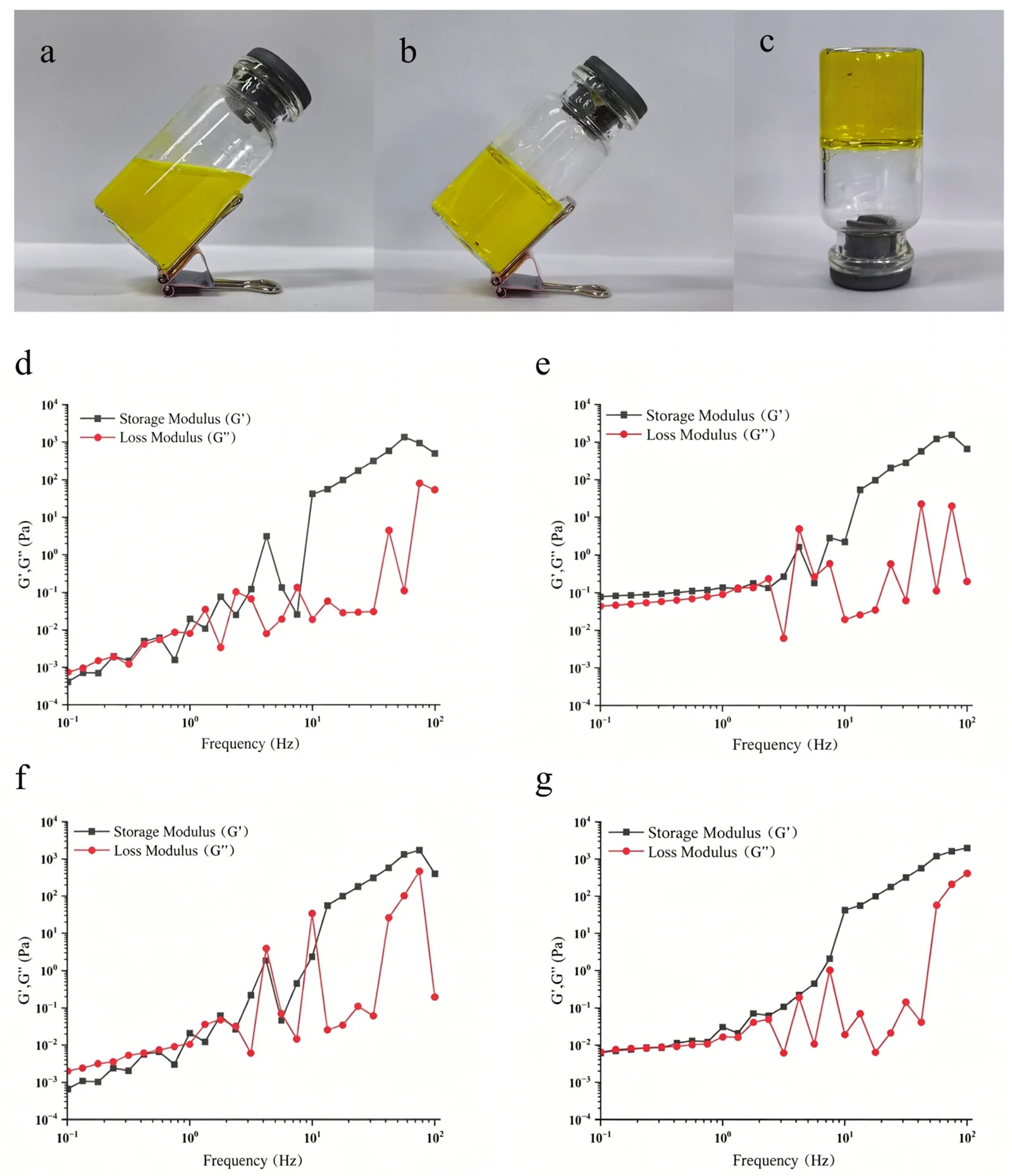
Inversion tests and rheological measurement verify the successful sol–gel transition and viscoelastic property of GB self-assembled hydrogel. (**a**) Formation of GB self-assembled hydrogel before gelation observed by the inverted method. (**b**) Formation after gelation observed by the inverted method. (**c**) Formation after inversion of GB self-assembled hydrogel post-gelation observed by the inverted method. Dynamic frequency-scanning rheological curves of GB self-assembled hydrogels at different concentration ratios: (**d**) 6 mM:1.5 mM, (**e**) 6 mM:2 mM, (**f**) 12 mM:3 mM, and (**g**) 12 mM:4 mM.

With GA concentration fixed at 12 mM, the effect of BBR ratio was investigated. Results showed that BBR not only did not destroy the gel structure but also exerted an obvious synergistic assembly effect. Vial inversion tests (Figure 1a–c) showed that a stable self-assembled hydrogel could form even at 1 mM BBR and 3 mM GA. In addition, the system significantly improved the water solubility of BBR from 2 mg/mL to 5 mg/mL [23], suggesting that the amphiphilic structure of GA acted as a molecular self-assembly carrier, encapsulating and stably dispersing BBR via hydrophobic microdomains and overcoming its inherent solubility limitation.

##### Rheological Results

Frequency sweep results showed that all systems exhibited frequency-dependent viscoelastic behavior. Dynamic frequency sweep tests were conducted on GB hydrogels (Figure 1d–g). In the low-frequency region (0.1–1 Hz), G′ and G″ were low and close, indicating a loose structure. As frequency increased (>10 Hz), G′ gradually exceeded G″ in some systems, showing a transition from solution to gel state. Compared with the binary GB co-assembly systems, pure berberine (BBR) aqueous solution failed to form an elastic gel network and behaved as a typical liquid-like system, with G″ always higher than G′. Although single glycyrrhizic acid monoammonium salt (GA) could form a hydrogel at 12 mM, its storage modulus was relatively low, representing a mechanically weak network. The binary co-assembly of GA and BBR brought about synergistic non-covalent cross-linking effects and greatly improved the mechanical performance of the supramolecular network. Significant differences were observed among different ratios: in low-concentration or mismatched systems (e.g., 6 mM GA:1.5 mM BBR, 6 mM GA:2 mM BBR), G′ was generally low and obviously fluctuated, with crossover between G′ and G″ in some frequency ranges, indicating insufficient intermolecular interactions and discontinuous network structure. In high-concentration systems (12 mM GA and 3 mM BBR, 12 mM GA and 4 mM BBR), G′ was significantly increased, indicating a well-developed network. Among them, the 12 mM:4 mM group showed optimal rheological properties: G′ was consistently higher than G″ across the entire frequency range, with a stable trend and no structural damage, confirming the formation of a uniform and stable 3D gel network. Therefore, 12 mM GA:4 mM BBR was determined as the optimal ratio. It should be noted that the moduli showed slight fluctuations and an upward trend at frequencies above 10 Hz, which may be partially attributed to the inertial effects of the instrument and edge effects at high shear rates. Therefore, the viscoelastic behavior in the 0.1–10 Hz range was used as the main basis for formulation evaluation, and the frequency sweep data were only used to characterize the gel viscoelasticity rather than to directly infer the absolute mechanical strength of the material.

Taking gel formation ability, macroscopic homogeneity, and rheological stability into comprehensive consideration, the formulation of 12 mM GA and 4 mM BBR was determined as the optimal ratio for subsequent experiments.

#### 2.1.2. Characterization Results of GB Hydrogel

##### TEM Results

**Figure 2:**
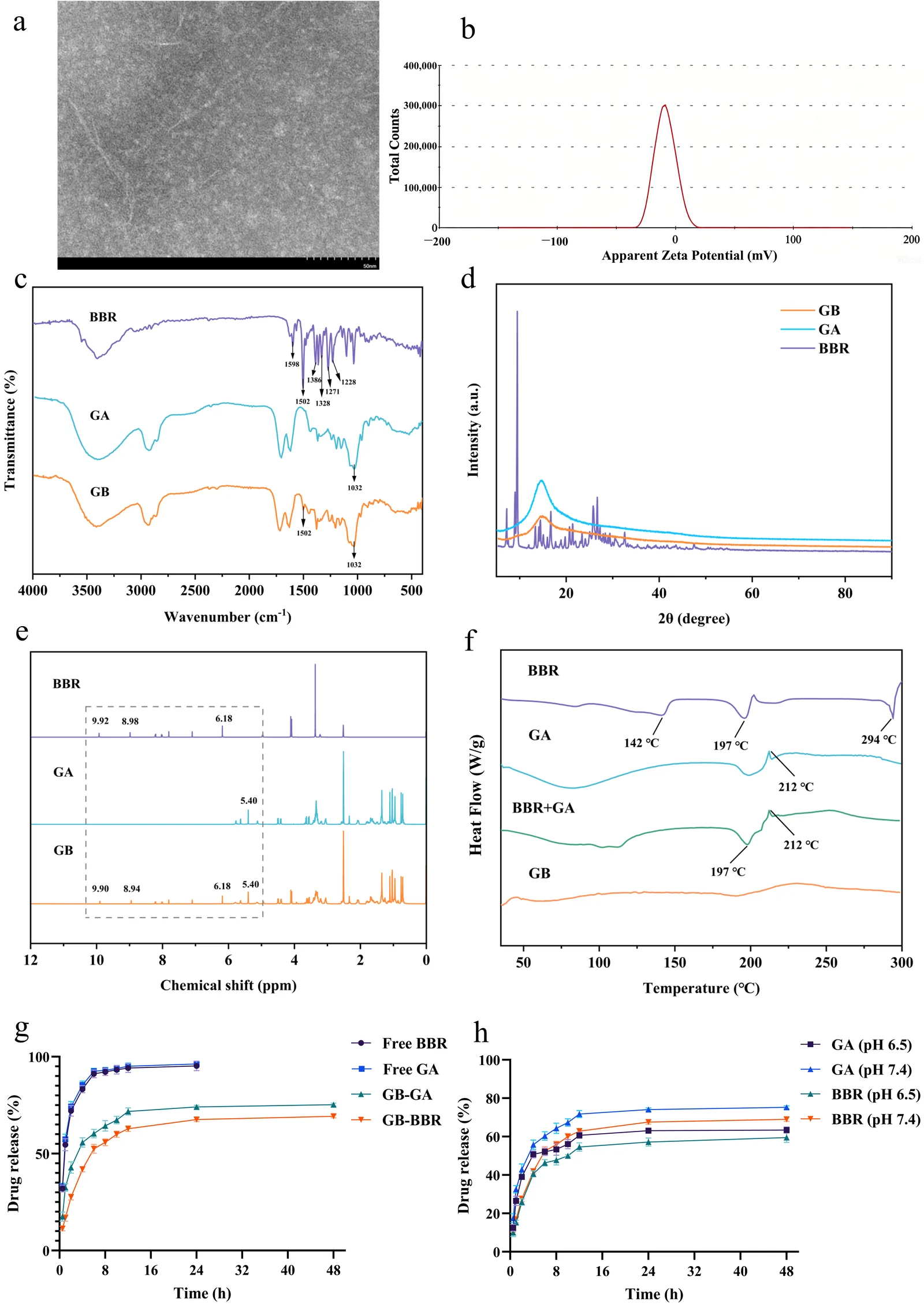
The microstructure, physicochemical properties, and in vitro drug release behavior of GB self-assembled hydrogel were characterized. (**a**) TEM images of GB self-assembled hydrogel (scale bar: 50 nm in the lower right corner). (**b**) Zeta potential values of GB self-assembled hydrogel. (**c**) Infrared spectra of BBR, GA, and GB self-assembled hydrogels. (**d**) XRD results of BBR, GA, and GB self-assembled hydrogels. (**e**) ^1^H-NMR data of BBR, GA, and GB self-assembled hydrogels. (**f**) DSC curves of BBR, GA, and GB self-assembled hydrogels. (**g**) Drug release profiles of free BBR, GA, GB, and GB self-assembled hydrogels at pH 7.4. (**h**) Drug release profiles of GA and BBR in GB self-assembled hydrogels under two different pH conditions.

Image analysis of multiple randomly selected visual fields from the TEM micrograph in Figure 2 was performed to quantify fiber dimensions. Statistical measurement of dozens of individual fibers confirmed that the single nanofiber had an average length of approximately 150 nm and uniform nanoscale diameter, interlacing to form a 3D network skeleton that provided excellent spatial structure for water and drug encapsulation. This nanofibrous network was consistent with the reported supramolecular nanofiber structure of GA self-assembly [24]. Recent studies have also showed that traditional Chinese herbal small molecules such as rhein could form similar hierarchical nanofibrous networks with GA mediated by metal ions, further verifying the universality of GA-based supramolecular self-assembly [25,26]. Non-covalent forces such as hydrogen bonding and π–π stacking dominated the self-assembly process. Image analysis confirmed that the single nanofiber had an average length of approximately 150 nm, interlacing to form a 3D network skeleton that provided an excellent spatial structure for water and drug encapsulation.

##### Zeta Potential

In this study, the GB hydrogel showed a stable surface potential of about −20 mV (Figure 2b), significantly higher than that of GA alone. Positively charged BBR increased the surface potential of negatively charged GA from −56.77 mV to −20 mV, which revealed intense electrostatic attraction existing between BBR and GA nanofibers. Meanwhile, the GB hydrogel exhibited a zeta potential of approximately −20 mV, which may generate moderate electrostatic repulsion within the supramolecular network and help alleviate the disordered aggregation of internal particles, partially supporting the favorable colloidal stability of the hydrogel system [27].

##### FT-IR Results

FT-IR was used to explore intermolecular interactions in the GB hydrogel (Figure 2c). The characteristic absorption peak of BBR at 1598 cm^−1^ corresponded to the stretching vibration of its quaternary ammonium ion (C=N+), and its intensity decreased in the GB spectrum, which could be attributed to electrostatic interactions between GA and BBR. In addition, the CN stretching vibration peaks of BBR at 1226, 1271, 1332, and 1388 cm^−1^ showed reduced intensity in the GB hydrogel. The peak at 1032 cm^−1^ related to COOH stretching and bending vibration also weakened in GB, suggesting that the movement of COOH was affected by BBR assembly. These findings further confirmed electrostatic interactions between GA (COOH) and BBR (C=N+). Notably, the aromatic C=C stretching peak of BBR at 1502 cm^−1^ was also significantly weakened. Comprehensive characterization including FT-IR, XRD, and ^1^H-NMR collectively indicated that hydrogen bonding, π–π stacking, and electrostatic interactions acted as key driving forces participating in the co-assembly of GB nanofibrous networks.

##### XRD Results

XRD was used to characterize the crystallographic features of BBR, GA, and the GB hydrogel (Figure 2d). BBR showed typical crystalline peaks at 2θ = 7.06°, 9.34°, 14.34°, 16.56°, 20.56°, 21.2°, 25.62°, 26.52°, and 32.38°, indicating a well-defined crystalline structure. However, these crystalline peaks completely disappeared in the GB hydrogel pattern, with only a broad amorphous diffuse peak around 20°, indicating amorphous particles. The loss of crystallinity resulted from spontaneous molecular rearrangement between GA and BBR involving multiple weak bonds (hydrogen bonds, electrostatic interactions), which weakened the peak at 14.65° observed in GA. XRD results further confirmed that significant intermolecular interactions occurred between BBR and GA in the GB hydrogel, transforming the original crystalline structure into an amorphous network, which was conducive to improving drug solubility and release performance.

##### ^1^H-NMR Results

^1^H-NMR was used to further investigate the molecular interaction mechanism of the self-assembled hydrogel (Figure 2e). In the ^1^H-NMR spectrum of BBR, aromatic proton signals at δ 9.92 ppm and 8.98 ppm were attributed to the isoquinoline skeleton, and a characteristic peak at δ 6.18 ppm belonged to the aromatic ring. In the GA spectrum, an obvious characteristic peak at δ 5.40 ppm was assigned to the characteristic proton of its glycoside structure, and multiple strong signals in the range δ 0.5–2.5 ppm corresponded to methyl and methylene protons of the GA triterpene skeleton. After BBR and GA formed the GB self-assembly system, characteristic signals of both components remained, but some peaks showed slight changes. The aromatic proton signals of BBR shifted from δ 9.92 ppm and 8.98 ppm to δ 9.90 ppm and 8.94 ppm, respectively, while the characteristic peak of GA at δ 5.40 ppm decreased in intensity. These slight chemical shifts indicated that no new covalent bonds were formed between BBR and GA, but weak intermolecular interactions (hydrogen bonds and π–π stacking) altered the chemical environment of some protons, consistent with the FT-IR and XRD results.

##### DSC Results

DSC was applied to characterize the thermal transition properties of pure BBR, GA, and GB composite hydrogel (Figure 2f). BBR showed obvious endothermic peaks at 142 °C and 197 °C, with 197 °C being a typical melting peak, indicating good crystallinity. GA showed an obvious exothermic peak at about 212 °C, corresponding to its characteristic thermal transition. In the DSC curve of the BBR+GA physical mixture, thermal peaks near 197 °C and 212 °C were observed simultaneously, indicating that the two components maintained their respective crystalline structures without obvious interactions. In contrast, the characteristic thermal peaks of BBR and GA were significantly weakened or even disappeared in the GB hydrogel curve, with gentle thermal behavior and only a weak broad endothermic signal in a wide temperature range. This indicated that significant intermolecular interactions occurred between BBR and GA during self-assembly, destroying or transforming the original crystalline structure into an amorphous state, thus altering the thermal transition characteristics.

Combined with the FT-IR, XRD, and ^1^H-NMR results, it could be inferred that nitrogen-containing structures in BBR might form hydrogen bonds and electrostatic interactions with carboxyl groups in GA, and π–π stacking might exist between the aromatic rings of BBR. These non-covalent interactions jointly drove the ordered aggregation of the two small molecules in the aqueous solution and further formed a stable 3D network structure.

#### 2.1.3. In Vitro Release Behavior

As shown in Figure 2g, free GA and BBR released rapidly at pH 7.4, with 24 h cumulative release rates of 96.58 ± 1.82% and 95.49 ± 0.45%, respectively, and almost complete release within 6 h, conforming to free diffusion. In contrast, the release rates of GA and BBR from the GB hydrogel were significantly slowed, with 24 h cumulative release rates of 75.32 ± 0.81% and 69.32 ± 0.82%, reaching a plateau at about 12 h, showing remarkable sustained-release behavior. This was attributed to the 3D network structure of the GB hydrogel: nanofibers formed by non-covalent interactions intertwined into a dense network, restricting the diffusion of active molecules to a certain extent, achieving sustained drug release [28].

In addition, the GB hydrogel exhibited obvious pH-dependent release behavior (Figure 2h). The release rates of GA and BBR were relatively low at pH 6.5 but significantly increased at pH 7.4. This pH-responsive release was attributed to the dissociation state of carboxyl groups in GA under different pH conditions [29]: at pH 7.4, the carboxyl groups in GA may become deprotonated and dissociated, which could enhance intermolecular electrostatic repulsion. This effect might swell the gel skeleton, increase porosity, loosen the compact self-assembled fiber network, and consequently facilitate drug diffusion under altered pH environments. The diabetic chronic wound microenvironment is usually weakly alkaline due to persistent inflammation and excessive exudate [30]. The release characteristics of the GB hydrogel at pH 7.4 endowed it with pH-dependent pathological microenvironment-responsive properties, expected to accelerate the release of high-concentration BBR for antibacterial efficacy and synchronously release GA for combined synergistic anti-inflammation and tissue repair during the most severe infection and inflammation stage.

#### 2.1.4. Cytotoxicity

**Figure 3:**
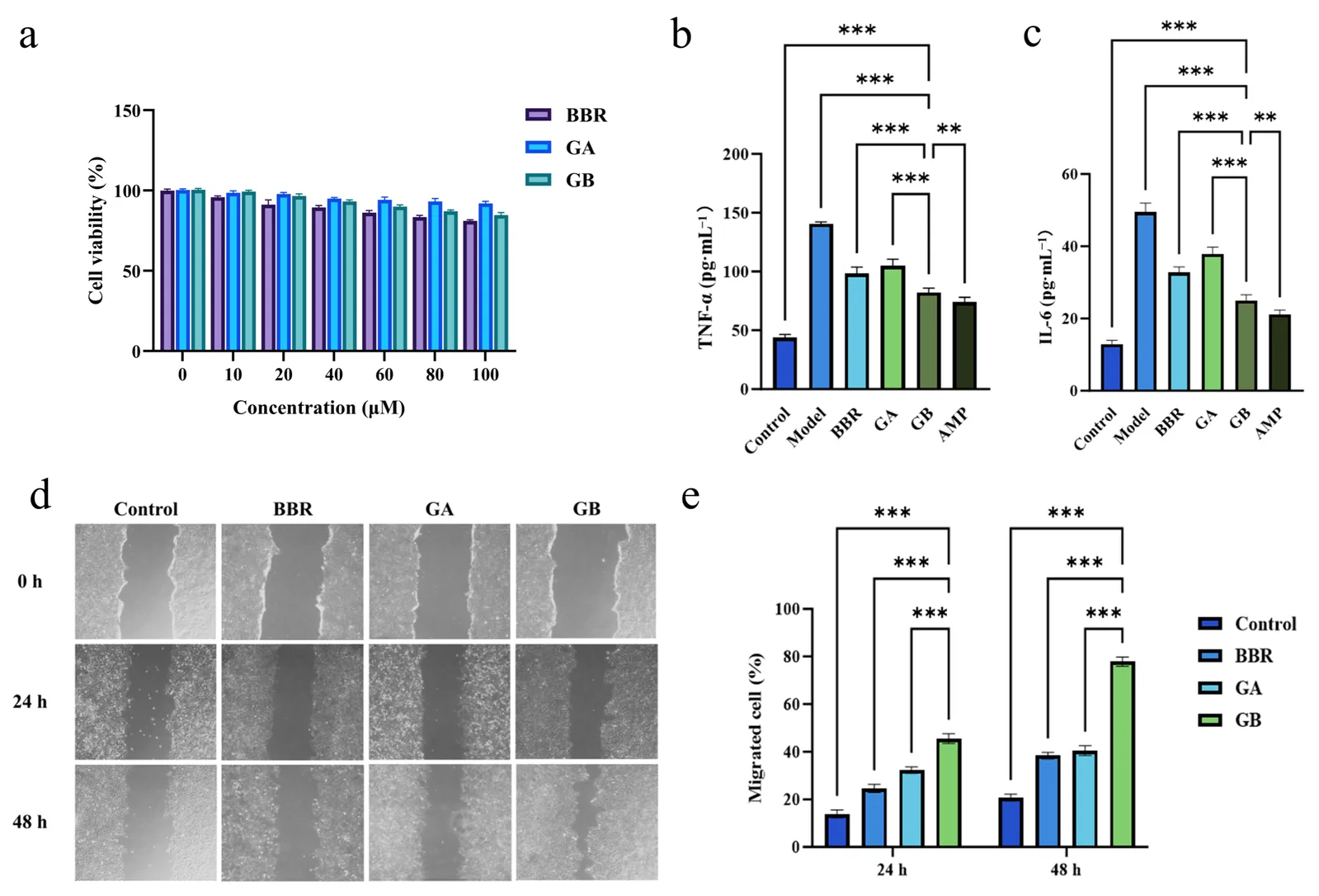
Cellular experiments confirm that GB is non-cytotoxic, anti-inflammatory, and effective at accelerating keratinocyte migration. (**a**) Effects of different drug concentrations on RAW264.7 cell viability after 24 h incubation. (**b**) TNF-α levels in cell supernatant after 24 h LPS stimulation. (**c**) IL-6 levels in cell supernatant after 24 h LPS stimulation. (**d**) Scratch assay images of HaCaT cells at 0 h, 24 h, and 48 h. (**e**) Quantitative analysis of scratch healing rate at 24 h and 48 h. The asterisks indicate that the differences are statistically significant. Specifically, ** indicates *p* < 0.01, and *** indicates *p* < 0.001.

Favorable biocompatibility serves as an essential precondition for hydrogel-based drug carriers in biomedical fields. The Cell Counting Kit-8 (CCK-8) assay was used to evaluate the in vitro cytotoxicity of GA, BBR, and the GB hydrogel on RAW264.7 cells. As shown in Figure 3a, after 24 h incubation at concentrations of up to 100 μM (for the GB hydrogel, expressed as the equivalent BBR concentration), GA maintained high cell viability (>95%), showing good biosafety; free BBR at the same concentration showed certain cell proliferation inhibition, with cell viability reduced. These results confirmed that the GB hydrogel possessed excellent in vitro biocompatibility.

#### 2.1.5. Anti-Inflammatory Effect

As illustrated in Figure 3b,c, in contrast to blank control samples, the LPS-challenged model group displayed markedly increased TNF-α and IL-6 concentrations, which verifies the successful construction of an inflammatory cell model. Among all treatment groups, the antibiotic AMP group exerted the strongest inhibitory effect on inflammatory factor expression. GA and BBR single treatments showed moderate anti-inflammatory activity with limited efficacy. Notably, the GB hydrogel group displayed significantly enhanced inhibitory effects on TNF-α and IL-6 expression compared with the single-agent groups, second only to the AMP group, indicating a combined anti-inflammatory effect of the two components. Similar synergistic anti-inflammatory strategies have been verified in other self-assembly systems [31]. For example, BBR-loaded ROS-responsive hydrogels could significantly reduce TNF-α and IL-6 levels in diabetic wounds by promoting macrophage polarization to M2 type [11], consistent with the anti-inflammatory results of this study.

#### 2.1.6. Cell Scratch Assay

The healing of diabetic chronic wounds is often affected by persistent inflammation, decreased cell migration, and impaired tissue regeneration. Keratinocyte migration is a key step in re-epithelialization during wound healing, and its migration ability directly affects wound closure speed [32]. According to the data presented in Figure 3d,e, compared to the control batch, all treatment groups showed improved cell migration rates to varying degrees. Among them, the GB hydrogel treatment group showed obvious cell migration at 24 h, and most scratch areas were covered by cells at 48 h, with the highest migration rate, showing a significant pro-migratory effect.

These results may stem from the inherent pharmacological activity of BBR and GA. Specifically, BBR could improve the cell growth microenvironment by regulating inflammation and oxidative stress, while glycyrrhizic acid compounds exert immunomodulatory and tissue repair effects [10,33,34]. Therefore, after self-assembly, GA and BBR may exert synergistic effects, promoting the superior migration-promoting effect of the GB hydrogel.

#### 2.1.7. Antibacterial Effect

**Figure 4:**
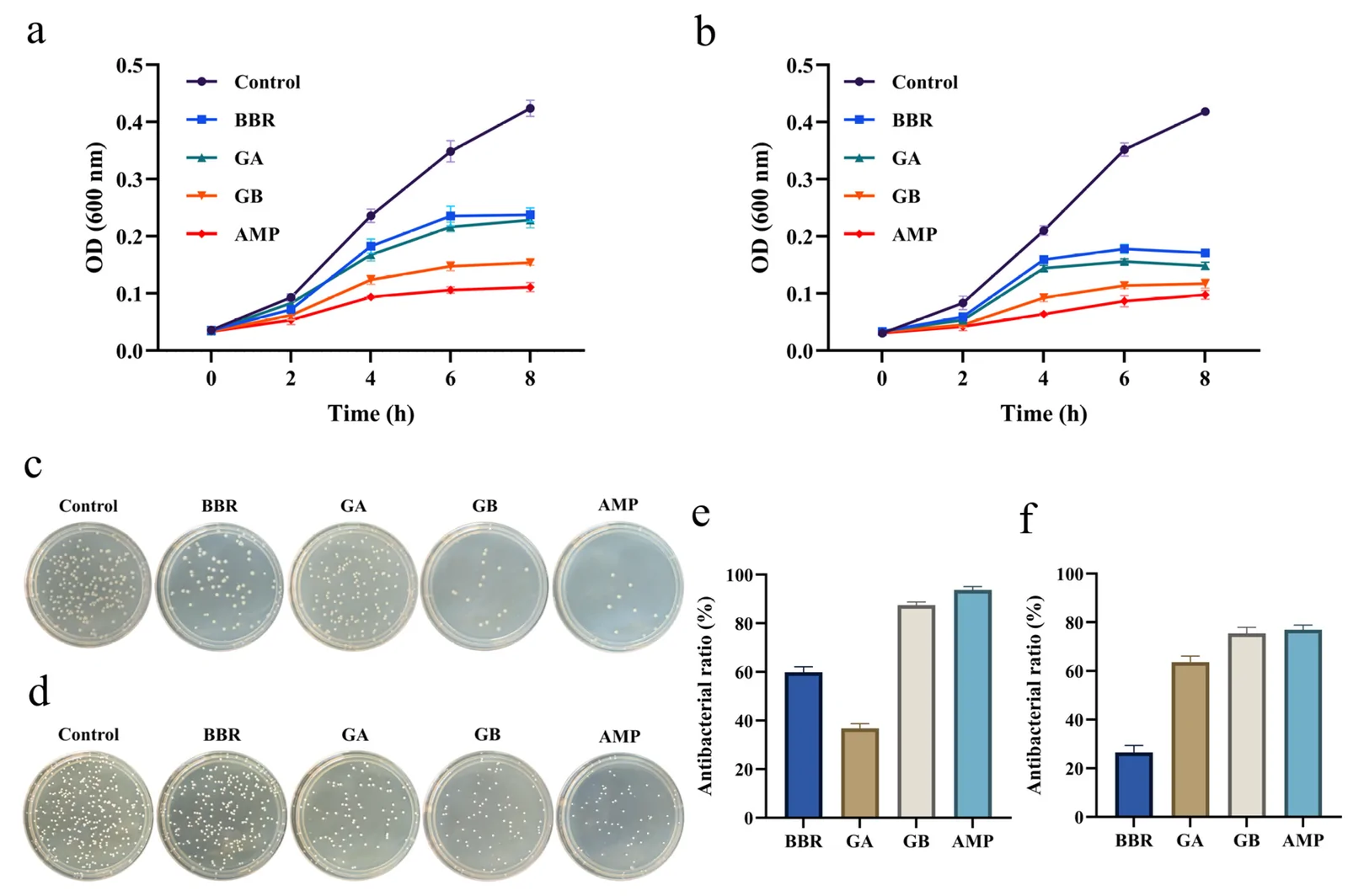
In vitro antibacterial assays verify the potent inhibitory effect of GB hydrogel on *E. coli* and *S. aureus*. (**a**) Growth curve of *E. coli*. (**b**) Growth curve of *S. aureus*. (**c**) Antibacterial inhibition profile on *E. coli* plates. (**d**) Antibacterial inhibition profile on *S. aureus* plates. (**e**) Inhibition rates of different treatment groups against *E. coli.* (**f**) Inhibition rates of different treatment groups against *S. aureus*.

AMP was used as a positive control. Bacterial growth curve results (Figure 4a,b) showed that bacteria both grew normally and entered the logarithmic phase rapidly in the control group, with significantly increased OD values over time. Following drug treatment, bacterial growth was inhibited to varying degrees in all treatment groups. As shown in Figure 4c,d, bacteria grew normally in the control group, while all treatment groups showed varying degrees of inhibition. For *E. coli*, BBR showed higher inhibition (~60%) than GA (~36%); for *S. aureus*, GA achieved better inhibition (~60%) than BBR (~25%), indicating complementary antibacterial spectra (Figure 4e,f).

After co-assembly into the GB hydrogel, the antibacterial ability was significantly enhanced, with inhibition rates against *E. coli* and *S. aureus* increased to ~88% and 70%, respectively, significantly higher than single-agent groups, confirming the enhanced and complementary antibacterial activity of the GB system. The antibiotic AMP group showed the strongest antibacterial ability as a positive control. These results were related to the antibacterial mechanisms of BBR and GA and the advantages of the self-assembled structure. BBR damages bacterial membranes and inhibits DNA replication and metabolism, while GA regulates membrane permeability/stability [35,36]. The self-assembly system improved dispersion and stability, increased bacterial contact, and achieved sustained release. GA and BBR complemented each other in antibacterial spectra, exerting strong inhibitory effects on both Gram-negative and Gram-positive bacteria, further verifying the potential of GB self-assembled hydrogel in the treatment of infected wounds.

#### 2.1.8. Hemolysis Assay

**Figure 5:**
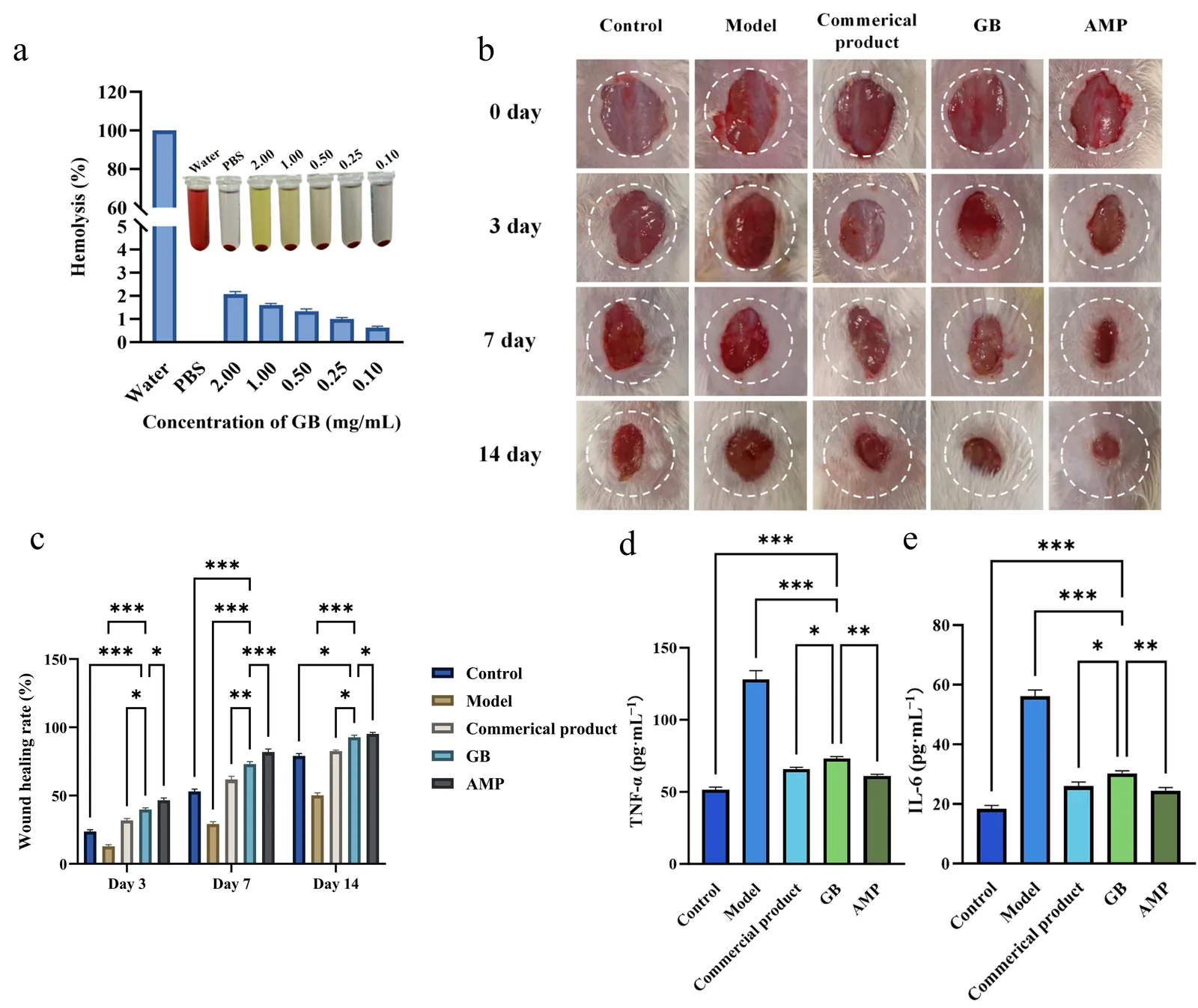
Histological staining quantitation reveals that GB hydrogel improves epidermal, granulation, and collagen regeneration in diabetic wounds. (**a**) Results of the hemolytic assay. (**b**) Wound healing progress at different time points (note: white circles have a diameter of 1 cm). (**c**) Wound healing rates at different time points. (**d**) Levels of TNF-α and (**e**) IL-6 immunological markers. The asterisks indicate that the differences are statistically significant. Specifically, * indicates *p* < 0.05, ** indicates *p* < 0.01, and *** indicates *p* < 0.001.

A hemolysis test was performed to assess the biosafety and biocompatibility of the self-assembled GB hydrogel. Distilled water served as the positive reference, while PBS buffer was set as the negative reference group. It can be seen from Figure 5a that the positive group exhibited a 100% hemolysis ratio, and zero hemolysis was detected for the negative control. The GB hydrogel showed no obvious hemolysis at all concentrations; even at 2 mg/mL, the hemolysis rate was still less than 2%, indicating excellent blood compatibility and meeting safety requirements (hemolysis rate <5% is considered non-hemolytic and safe for in vivo application) [37].

#### 2.1.9. Wound Healing Rate

To investigate the therapeutic efficacy of the GB hydrogel on wound regeneration, we generated a full-thickness skin lesion model using diabetic mice. Wound closure rates were calculated by measuring wound area changes. Quantitative results showed that wound area gradually contracted over time in all groups, but healing characteristics varied greatly (Figure 5b).

As illustrated in Figure 5c, compared with the diabetic blank model group, all treatment groups demonstrated increased wound healing. On day 7, the healing rate of the blank group was only 29.17%, while that of the GB hydrogel group reached 73.10%, significantly better than the commercial hydrogel group (61.83%). On day 14, the GB hydrogel group still achieved high-quality tissue reconstruction. These results indicated that the GB hydrogel could significantly accelerate diabetic wound healing.

Notably, the GB hydrogel group showed excellent therapeutic effects in promoting wound healing, consistent with the reported pro-healing effects of licorice composite gels [38]. On the one hand, BBR and GA both showed certain anti-inflammatory and antibacterial activities, inhibiting inflammatory factor release and reducing bacterial infection locally, improving the wound microenvironment; on the other hand, the 3D network structure formed by the self-assembled hydrogel created a moist protective environment on the wound surface and maintained stable local drug concentration through sustained release of active components, facilitating wound treatment.

#### 2.1.10. In Vivo Anti-Inflammatory Effect by ELISA

ELISA measurements were carried out to detect the expression amounts of TNF-α and IL-6 within wound tissues harvested from experimental mice. Data from Figure 5d,e revealed obvious upregulation of the two inflammatory mediators in diabetic model animals versus the normal control batch, which signals robust inflammatory infiltration inside skin wounds. Upon hydrogel intervention, GB treatment and commercial hydrogel treatment both achieved remarkable downregulation of cytokine levels, with final values of around 72 pg/mL and 34 pg/mL, respectively. The statistical gap between hydrogel-treated groups and the model group was extremely significant, providing solid evidence that GB self-assembled hydrogel could efficiently relieve excessive inflammatory reactions in wound regions.

#### 2.1.11. Wound Healing Evaluation by H&E Staining

**Figure 6:**
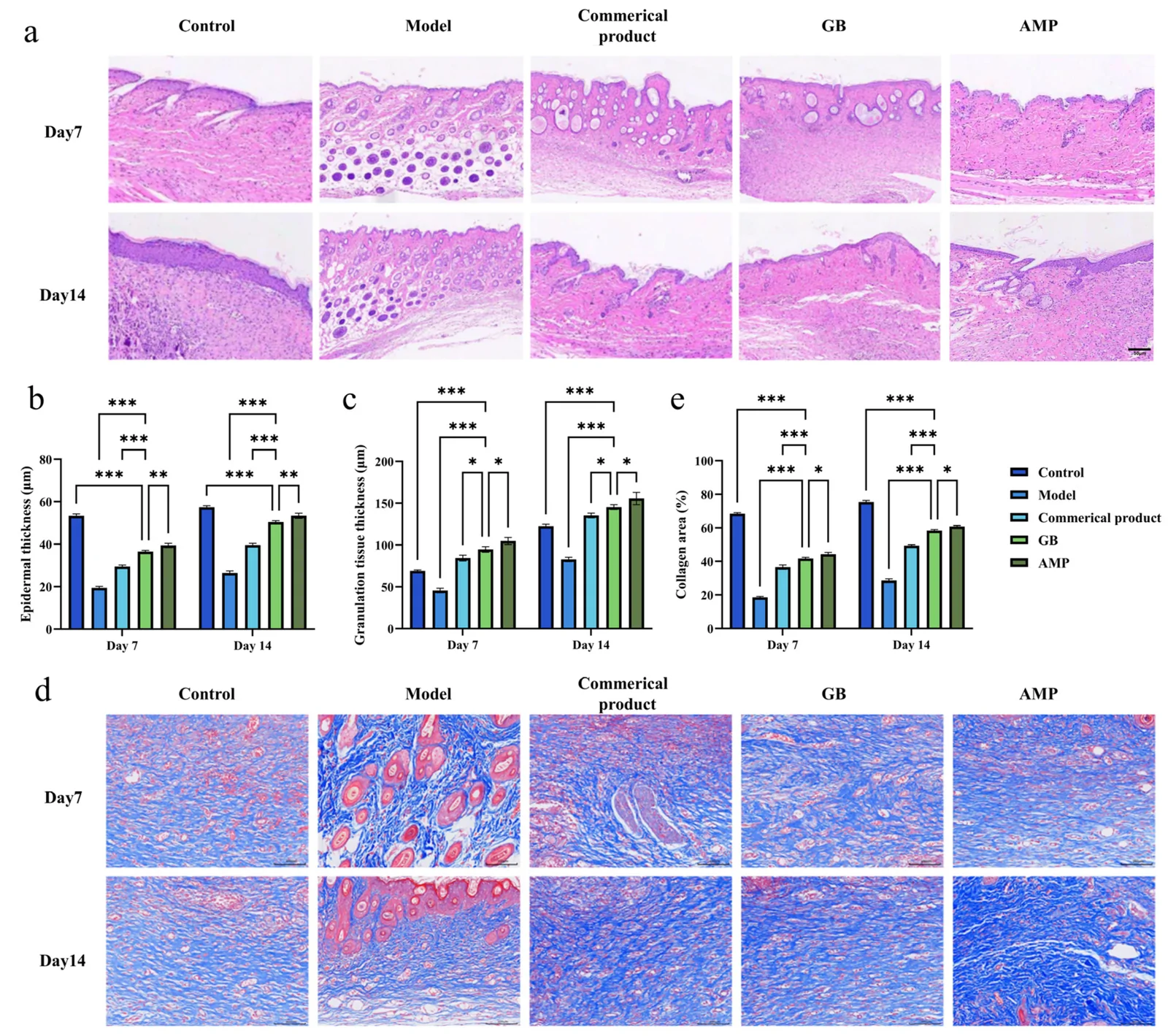
Histological staining quantitation reveals that GB hydrogel improves epidermal, granulation, and collagen regeneration in diabetic wounds. (**a**) Histopathological changes in wound tissues of mice after drug intervention as observed by H&E staining (scale bar: 50 μm). (**b**) Quantitative analysis of epidermal thickness by H&E staining. (**c**) Quantitative analysis of granulation tissue thickness by H&E staining. (**d**) Masson staining images of wound tissues from diabetic mice at different healing time points across groups (scale bar: 100 μm). (**e**) Quantitative analysis of collagen deposition in wound tissues of diabetic mice across groups. The asterisks indicate that the differences are statistically significant. Specifically, * indicates *p* < 0.05, ** indicates *p* < 0.01, and *** indicates *p* < 0.001.

H&E staining was used to evaluate wound tissue repair. As shown in Figure 6a, the diabetic model group showed obvious tissue defects and severe inflammatory cell infiltration on days 7 and 14, with low epidermal thickness and granulation tissue thickness (Figure 6b,c). In contrast, the GB hydrogel treatment group exhibited significantly improved wound reepithelialization; by day 14, a continuous layer of epidermal cells had formed, with well-developed and regularly arranged granulation tissue. Both the epidermal thickness (~50 μm) and granulation tissue thickness (~145 μm) were markedly superior to those observed in the model group and the commercially available hydrogel group.

#### 2.1.12. Wound Healing Evaluation by Masson Staining

Extracellular matrix remodeling and orderly collagen deposition are core pathological indicators for evaluating wound healing quality and mechanical recovery. As shown in Figure 6d,e, the model group showed sparse and broken collagen, with a collagen area fraction of only 28.66 ± 0.90% on day 14. Collagen deposition was enhanced to different extents in each treatment group. Among these groups, the GB hydrogel group exhibited the optimal therapeutic outcome. On day 14, the collagen area fraction of this group was 58.01 ± 1.17%. This value was remarkably higher than that of the model group and also exceeded the value of the commercial hydrogel group (49.00 ± 1.12%). These results demonstrated that the GB hydrogel possessed a strong capacity to facilitate collagen synthesis and deposition.

#### 2.1.13. Angiogenesis Evaluation by CD31 and α-SMA Analysis

**Figure 7:**
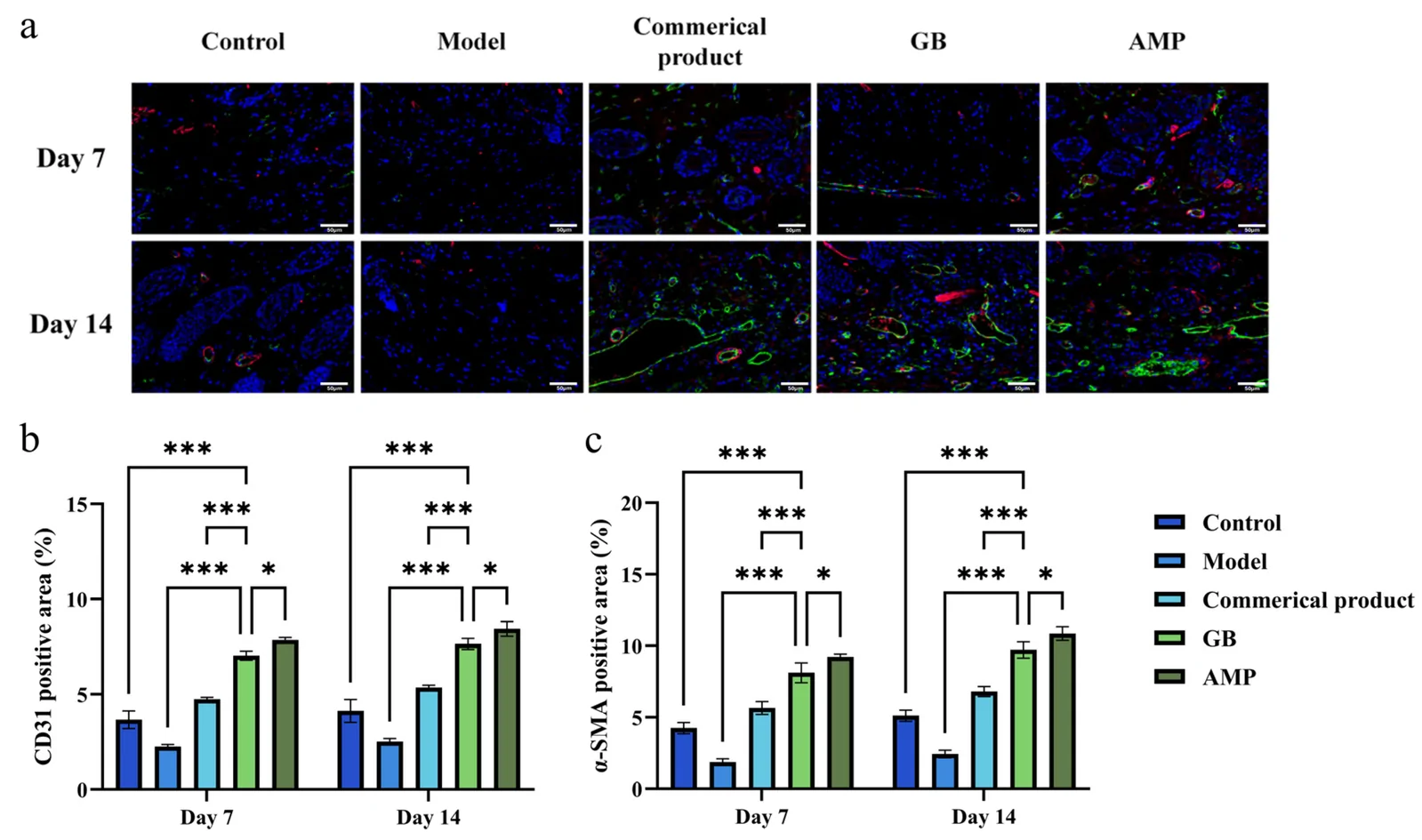
Evaluation of angiogenesis in GB hydrogels. (**a**) Immunofluorescence dual staining of CD31 and α-SMA in wound tissues from diabetic mice across groups (red indicates CD31-positive expression, green indicates α-SMA-positive expression, blue represents nuclei stained with DAPI) (scale bar: 50 μm). (**b**) Quantitative analysis of the proportion of CD31-positive areas in diabetic wounds across groups. (**c**) Quantitative analysis of the proportion of α-SMA-positive areas in diabetic wounds across groups. The asterisks indicate that the differences are statistically significant. Specifically, * indicates *p* < 0.05, and *** indicates *p* < 0.001.

CD31 and α-SMA immunofluorescence double staining was used to evaluate neovascularization in wounds (Figure 7a). Compared with the model group, the Commercial product, GB, and AMP groups exhibited enhanced CD31 and α-SMA expression, particularly on day 14, with more mature, vessel-like structures observed in the GB and AMP groups. As shown in Figure 7b,c, the model group showed extremely low positive expression areas of CD31 and α-SMA on day 14 (less than 3%). The GB hydrogel group showed excellent pro-microvascular network construction ability as early as day 7 (CD31 expression 7.26%, α-SMA expression 8.13%). On day 14, CD31 expression in the GB group further increased to 8.11% and α-SMA expression to 9.68%, with high co-localization, indicating that the GB hydrogel could not only promote neovascularization but also enhance the maturation and stability of microvessels. Notably, BBR-loaded ROS-scavenging hydrogels were also confirmed to promote mature neovascularization in diabetic wounds through multimodal therapy [39], consistent with the vascularization effect of the GB hydrogel in this study.

### 2.2. Discussions

In this study, a carrier-free binary supramolecular GB hydrogel was successfully fabricated via non-covalent co-assembly of berberine (BBR) and monoammonium glycyrrhizinate (GA). Multiple characterizations including TEM, FT-IR, ^1^H-NMR, XRD, and DSC collectively demonstrated that hydrogen bonding, electrostatic interaction, and π–π stacking synergistically drove the formation of uniform nanofibrous three-dimensional networks. Compared with single-component GA hydrogels reported in the existing literature, the introduction of BBR did not disrupt gel-network integrity, but achieved a complementary antibacterial spectrum and reinforced anti-inflammatory capacity without additional polymer cross-linkers or exogenous drug carriers. Most reported berberine-loaded wound dressings rely on exogenous polymer matrices for drug encapsulation, which may bring potential biocompatibility risks and burst drug release. By contrast, our GB system realizes in situ loading of BBR through supramolecular co-assembly, avoiding extra carrier-related safety concerns.

In vitro experiments confirmed that the GB hydrogel possessed favorable cytocompatibility. It could suppress over-expression of pro-inflammatory TNF-α and IL-6 in LPS-stimulated RAW264.7 macrophages, accelerate HaCaT keratinocyte migration, and exert synergistic antibacterial effects against *S. aureus* and *E. coli*. In vitro release assays revealed obvious pH-responsive release behavior: a higher drug release rate was observed under pH 7.4 conditions. We speculated that de-protonation of carboxyl groups on GA molecules would enhance intermolecular electrostatic repulsion, loosen nanofiber networks, and consequently accelerate drug diffusion. However, this proposed molecular-level inference still needs further targeted experimental verification.

In vivo results demonstrated that the GB hydrogel could ameliorate the local inflammatory microenvironment, boost angiogenesis and collagen deposition, and remarkably accelerate full-thickness infected diabetic wound healing. Although our in vitro and in vivo results support its beneficial wound-repair performance, the exact downstream signaling pathways mediating anti-inflammatory and pro-repair effects remain unclear. At present, our evidence is mainly obtained from phenotypic observation and detection of several representative cytokines, lacking deeper mechanistic exploration at a gene or protein level.

It is worth noting that persistent inflammation, bacterial colonization, insufficient angiogenesis, and disordered collagen remodeling are not exclusive pathological hallmarks of diabetic ulcers. These pathological features are also frequently observed in other intractable chronic wounds induced by chronic venous insufficiency, chronic kidney disease, and autoimmune-related skin lesions. Benefiting from its integrated functions of broad-spectrum antibacterial activity, anti-inflammation, and promotion of cell regeneration, the GB supramolecular hydrogel shows potential applicability for these non-diabetic chronic wounds. Nevertheless, such extrapolation is only inferred from shared pathological characteristics; dedicated pre-clinical animal models are still required to verify its actual therapeutic efficacy for these different wound microenvironments. Collectively, this work enriches the construction strategy of herbal small-molecule carrier-free supramolecular biomaterials and provides experimental support for developing multifunctional wound dressings.

## 3. Conclusions

In the present work, we constructed a carrier-free supramolecular GB hydrogel via one-step non-covalent co-assembly of BBR and GA. A series of physicochemical characterizations verified the formation of nanofibrous hydrogel networks driven by hydrogen-bonding, electrostatic, and π–π stacking interactions. The GB hydrogel exhibited good biocompatibility, pH-responsive sustained release, and synergistic anti-bacterial and anti-inflammatory activities. Both in vitro cell assays and the in vivo infected diabetic wound model confirmed that the GB hydrogel could mitigate excessive local inflammation, promote angiogenesis and collagen deposition, and effectively accelerate chronic wound repair.

Nevertheless, several important limitations should be acknowledged in this study. First, we only detected several key inflammatory cytokines to evaluate biological effects, and the precise downstream molecular signaling pathways modulated by the GB hydrogel have not been fully illustrated. Second, long-term in vivo biosafety assessments including repeated-dose local irritation and major-organ systemic toxicity are absent. Third, comprehensive multi-disease pre-clinical evaluation covering various non-diabetic chronic wound models has not been completed.

For future investigations, multiple research directions deserve further exploration. Firstly, targeted mathematical models will be established to quantitatively interpret the drug-release mechanism of this co-assembled hydrogel, so as to realize accurate prediction and dynamic regulation of release performance. Secondly, we will further explore the intrinsic correlation between GA de-protonation behavior and intermolecular electrostatic repulsion, to clarify whether GA de-protonation can effectively amplify electrostatic repulsion and identify key molecular driving factors that govern hydrogel stability, assembly morphology, and release functions. Moreover, deeper mechanistic studies and systematic long-term biosafety evaluation, as well as efficacy verification on various chronic wound animal models, will be implemented to further advance the translational prospect of the GB hydrogel as a novel wound-repair dressing.

## 4. Materials and Methods

### 4.1. Materials and Reagents

BBR (purity ≥ 98%) and GA (purity ≥ 98%) were purchased from Shanghai Macklin Biochemical Co., Ltd. (Shanghai, China). Dulbecco’s modified eagle medium (DMEM), fetal bovine serum (FBS), trypsin, and penicillin-streptomycin solution were sourced from Beyotime Biotechnology (Shanghai, China). Phosphate-buffered saline (PBS) was purchased from Beijing Lanjieke Technology Co., Ltd. (Beijing, China). CCK-8, TNF-α, IL-6, and enzyme-linked immunosorbent assay (ELISA) kits were sourced from Beyotime Biotechnology (Shanghai, China). Lipopolysaccharide (LPS), streptozotocin (STZ), hematoxylin and eosin (H&E) staining solution, and Masson staining solution were supplied by Solarbio Science & Technology Co., Ltd. (Beijing, China). *Escherichia coli* (*E. coli*) and *Staphylococcus aureus* (*S. aureus*) strains were procured from Qingdao Haibo Biotechnology Co., Ltd. (Qingdao, China). Commercial hydrogel dressing was provided by Weihai Wego Yourui Bio-Materials Co., Ltd. (Weihai, China). Ampicillin (AMP, purity ≥ 98%) was acquired from Shanghai Macklin Biochemical Co., Ltd. (Shanghai, China). Tryptic soy broth (TSB), tryptic soy agar (TSA), and Luria-Bertani (LB) medium were supplied by BD Difco (Franklin Lakes, NJ, USA) for all microbiological assays. The remaining chemical reagents used in this experiment reached analytical grade.

### 4.2. Cells and Animals

The murine macrophage RAW264.7 cell line and human immortal epidermal keratinocyte HaCaT cells were acquired from Zhejiang Baidi Biotechnology Co., Ltd. (Shanghai, China), with official cell authentication certificates provided by the supplier. Mycoplasma contamination detection was performed by PCR before cell experiments, and only mycoplasma-negative cells were adopted for subsequent tests. Cells at passages 3–8 were used uniformly for all in vitro functional assays to avoid phenotypic drift caused by excessive passaging. All cells were maintained with standard culture protocols using DMEM containing 10% FBS and 1% penicillin-streptomycin mixture, incubated at 37 °C under humidified air with 5% CO_2_.

The Laboratory Animal Center of Heilongjiang University of Chinese Medicine provided 6-week-old female BALB/c mice (body mass: 20 ± 2 g). All animals were raised in a specific pathogen-free (SPF) animal room at constant temperature (22 ± 2 °C) and humidity (50% ± 5%), with a 12 h light/12 h dark alternating cycle. Each cage housed 4 mice at most, and all animals received a 7-day acclimatization feeding period before formal modeling to relieve any stress response. Female BALB/c mice (body mass: 20 ± 2 g) were used in this study. The T-cell immunodeficiency characteristic of mice can avoid the interference of individual immune differences on the inflammatory response, which helps to more stably evaluate the direct anti-inflammatory and pro-healing effects of the hydrogel material itself; it is also conducive to the establishment of a standardized infected wound model with good reproducibility. Prior to implementation, all animal trials obtained ethical certification from the Animal Ethics Committee of Heilongjiang University of Chinese Medicine (No. HUCM-2024-0315, approval issued 15 March 2024). All experimental procedures were carried out in strict accordance with national standards for the breeding and experimental utilization of laboratory animals.

### 4.3. Methods

#### 4.3.1. Preparation of GB Self-Assembled Hydrogel

After accurate weighing, BBR powder was dispersed into deionized water and continuously stirred at 70 °C over 10 min to achieve full solvation. Multiple BBR aqueous solutions with concentrations of 1, 1.5, 2, 3, and 4 mM were prepared and kept warm for subsequent utilization. Separately, an appropriate amount of GA powder was added to deionized water and dissolved with ultrasonic cleaner (KQ-500DE, Kunshan Ultrasonic Instrument Co., Ltd., Kunshan, China) until no obvious coagulation was observed, then stirred at 600 rpm in a 70 °C water bath for 10 min to obtain a colorless and transparent GA solution at different concentrations (3, 4, 6, 8, 10, 12, 14 mM). BBR solution was added dropwise to an equal volume of GA solution with continuous stirring at 1000 rpm and 70 °C. After complete mixing, the mixture was transferred to room temperature (25 °C) and stood still for 30 min to fully complete sol–gel transition and form uniform GB self-assembled hydrogel (Scheme 1). Combining vial inversion gel identification and rheological G′ comparison among all tested concentration gradients, the formulation of 12 mM GA and 4 mM BBR showed stable gel state and the highest storage modulus; it was thus selected as the optimal gelation ratio. Gelation was evaluated by the vial inversion method at room temperature (25 °C). The formulation optimization was based on three objective criteria in sequence: (1) successful gel formation (no flow within 30 s after vial inversion); (2) homogeneous appearance without phase separation or precipitation; (3) optimal viscoelastic properties (storage modulus G′ consistently higher than loss modulus G″ over the entire frequency range, with higher G′ value preferred). Each formulation was prepared independently for at least 3 batches to verify reproducibility. The gelation time of the optimal formulation (12 mM GA and 4 mM BBR) was approximately 18 ± 2 min at 25 °C, and the final pH of the hydrogel was 6.8 ± 0.1. The as-prepared hydrogel sample was pre-frozen at −80 °C over 12 h, followed by lyophilization for 48 h using a vacuum freeze dryer (Model ZLGJ-18, Ningbo Scientz Biotechnology Co., Ltd., Ningbo, China). The acquired lyophilized GB hydrogel powder was reserved for subsequent material characterizations. Prior to subsequent cell and animal experiments, the pre-lyophilized GB hydrogel solution was sterilized by ultraviolet irradiation for 30 min in a biosafety cabinet under aseptic conditions; all lyophilized hydrogel powder was weighed and re-dissolved using aseptic pipettes and sterile PBS in laminar flow hood for follow-up administration.

#### 4.3.2. Characterization of GB Hydrogel

##### Rheological Measurements

The rheological characteristics of specimens were investigated with a rotational rheometer (MARS60, HAAKE, Karlsruhe, Germany) fitted with a parallel-plate geometry possessing a diameter of 60 mm, with the measurement clearance fixed at 1 mm. A sufficient quantity of GB hydrogel was loaded onto the testing platform, followed by trimming away superfluous material; a thin coating of dimethyl silicone oil was then spread over the sample surface to inhibit water loss. Prior to frequency sweep tests, a strain amplitude sweep was performed at 1 Hz to determine the linear viscoelastic (LVE) range, and a strain value of 1% within the LVE region was selected for subsequent tests. After loading the sample, an equilibration time of 5 min was set to allow stress relaxation and temperature stabilization. Frequency sweep measurements ranging from 0.1 Hz to 100 Hz were performed at a constant temperature of 37 °C to acquire the frequency-dependent profiles of storage modulus (G′) and loss modulus (G″).

##### Transmission Electron Microscopy (TEM)

Transmission electron microscopy (TEM) was used to characterize the microstructure and network structure of the GB hydrogel. An appropriate amount of the diluted and pretreated GB hydrogel sample was dispensed onto the surface of a TEM copper grid substrate coated with a carbon support film and left to adsorb for 30 s to ensure that the hydrogel components were fully attached to the carbon support film. After adsorption was complete, excess liquid on the surface was carefully blotted away along the edges of the copper mesh using ash-free filter paper. During this process, direct contact between the filter paper and the sample observation area was avoided to prevent damage to the thin sample layer formed by adsorption. The prepared copper mesh sample was allowed to air-dry at room temperature for 20 min; to thoroughly remove residual moisture from the sample and eliminate the risk of structural distortion and instrument chamber contamination caused by moisture evaporation in the high-vacuum environment of the electron microscope, the sample was further dried in a vacuum oven for 12 h. Once the entire sample preparation process was complete, the copper mesh sample was loaded into the transmission electron microscope, the accelerating voltage was set to 200 kV, the microscopic network morphology of the GB hydrogel was observed, and electron microscope images were acquired for subsequent analysis.

##### Zeta Potential Measurement

GB hydrogel lyophilized powder was re-dissolved in deionized water to an appropriate concentration. A measure of 1 mL of the solution was injected into the potential cell and equilibrated for 120 s. Zeta potential values were determined on a Zeta potential and particle size analyzer (Zetasizer Nano ZS90, Malvern Panalytical, Malvern, UK).

##### Fourier Transform Infrared Spectroscopy (FT-IR)

A small amount of GA, BBR raw material, and GB hydrogel lyophilized powder were accurately weighed and fully ground with dry KBr powder in an agate mortar. The mixture was pressed into a translucent tablet at 80 MPa for 1–2 min. Using a pure KBr tablet as reference, FT-IR spectra were collected with a spectrometer (Vertex 70, Bruker, Ettlingen, Germany) with a wavenumber range of 400–4000 cm^−1^.

##### X-Ray Diffraction (XRD)

The lyophilized powder derived from the GB hydrogel was characterized via X-ray diffractometry (D8-Advance, Bruker, Ettlingen, Germany). Testing conditions: Cu Kα radiation (λ = 0.154 nm), voltage 40 kV, current 40 mA, scanning range 2θ = 5–60°, scanning rate 10°/min.

##### ^1^H-Nuclear Magnetic Resonance (^1^H-NMR)

A measure of 5.0 mg each of GA, BBR raw material, and GB hydrogel lyophilized powder were dissolved in 600 μL DMSO-d_6_ and transferred to a standard NMR tube. Tetramethylsilane (TMS) served as the internal reference. A nuclear magnetic resonance spectrometer (AVANCE III 400 MHz, Bruker, Ettlingen, Switzerland) was employed to collect ^1^H-NMR spectra at 37 °C.

##### Differential Scanning Calorimetry (DSC)

Approximately 5 mg each of GA, BBR, a physical mixture of GA and BBR, and GB hydrogel lyophilized powder were weighed and sealed in standard aluminum crucibles, with an empty aluminum crucible of the same specification as the reference. Under a dynamic, high-purity nitrogen atmosphere (with the gas flow rate set to 20 mL/min), the samples were subjected to a programmed temperature ramp at a heating rate of 10 °C/min over a temperature range of 0–300 °C, and the thermal analysis curves for each sample were continuously recorded and output. All measurements were conducted on a DSC instrument (214 Polyma, Netzsch, Selb, Germany).

### 4.4. High-Performance Liquid Chromatography (HPLC) Conditions

HPLC (2695, Waters, Milford, MA, USA) was adopted for sample analysis. A Diamonsil C18 column (4.6 mm × 250 mm, 5 μm) was used to conduct chromatographic separation, and column temperature was held constant at 25 °C. Methanol–water–glacial acetic acid (90:9.5:0.5, *v*/*v*/*v*) was used for isocratic elution at a flow rate of 0.8 mL/min. Sample injection volume and detection wavelength were set as 10 μL and 340 nm, respectively. The results of the methodological investigation were presented in the Supplementary Materials.

### 4.5. In Vitro Release Study

Raw GA and BBR powders were dissolved in Phosphate buffer saline (PBS) to prepare free drug groups. The release medium for free drugs was PBS (pH 7.4) supplemented with 0.5% Tween 80. In terms of the GB self-assembled hydrogel group, 20 mL of PBS solutions containing 0.5% Tween 80 at pH values of 6.5 and 7.4 were utilized as release media. 2 mL of the freshly prepared GB hydrogel (containing 12 mM GA and 4 mM BBR) was accurately loaded into dialysis bags with a molecular weight cutoff of 3500 Da, then immersed in 20 mL of release medium at 37 °C, and immersed in pH-matched PBS solution under continuous shaking at 100 rpm. At predetermined intervals (0.5, 1, 2, 4, 6, 8, 10, 12, 24 h), 1 milliliter of dialysate was collected, followed immediately by replenishing an equal amount of fresh PBS. HPLC was used to quantify the in vitro release contents of GA and BBR. The cumulative release rate was calculated using the following formula:

(1) $$E_{r}\;=\;\frac{V_{e}\sum_{1}^{n-1}C_{i}+\;V_{0}C_{n}}{m_{\mathrm{drug}}}\;$$

where $E_{r}$ represents the cumulative drug release rate; $V_{e}$ stands for the replacement volume of release medium; $V_{0}$ denotes the initial volume of release medium; $C_{i}$ indicates the drug concentration measured at the i-th sampling point; $C_{n}$ refers to the drug concentration detected at the n-th sampling point; $m_{drug}$ corresponds to the total mass of GA or BBR contained within the hydrogel, respectively.

### 4.6. Cell Culture

Prior to cell resuscitation, the complete culture medium was preheated to 37 °C and balanced at 25 °C. Cryopreserved cell vials were retrieved from liquid nitrogen and immediately thawed thoroughly within 2 min by continuous shaking in a 37 °C water bath. Under a biosafety cabinet (HFsafe-1200, Heal Force, Shanghai, China), the cell suspension was transferred into a centrifuge tube containing pre-added complete medium and blended gently. The mixture was centrifuged using a high-speed refrigerated centrifuge (TDL-16B, Shanghai Anting Scientific Instrument Factory, Shanghai, China) at 1000 rpm for 5 min, followed by the removal of cryoprotectant-containing supernatant. The cell pellet was resuspended in 1 mL of fresh medium via pipetting to form a single-cell suspension, which was seeded into a culture flask and incubated horizontally in a 5% CO_2_ incubator (HERAcell 240, Thermo Fisher Scientific, Waltham, MA, USA) at 37 °C. After 12 h of continuous culture, the entire medium was renewed, and cell adhesion and confluence status were visualized using an inverted microscope (CKX53, Olympus, Tokyo, Japan). RAW264.7 murine macrophages and HaCaT keratinocytes were maintained separately in complete DMEM supplemented with 10% (*v*/*v*) FBS and 1% penicillin-streptomycin (100 U/mL penicillin, 100 μg/mL streptomycin). All cells were incubated in a humidified incubator at 37 °C with 5% CO_2_. Cells were subcultured at 70–80% confluence using 0.25% trypsin-EDTA solution; trypsin digestion was terminated with an equal volume of complete medium. Only cells at passages 3–8 with negative mycoplasma PCR test results were used for all functional assays to avoid phenotypic drift. Medium was replaced every 48 h during routine expansion. RAW264.7 cells were seeded into 96-well plates at a density of 2 × 10^3^ cells per well for the CCK-8 test; RAW264.7 cells were seeded at 2 × 10^5^ cells/mL for LPS inflammatory model construction; HaCaT cells were seeded at 1 × 10^5^ cells/mL in 6-well plates for scratch migration detection.

### 4.7. Cytotoxicity Assay

A total volume of 100 μL RAW264.7 cell suspension was seeded into each well of 96-well plates at a fixed density of 2 × 10^3^ cells per well, with five parallel replicate wells arranged for every test group. The cells underwent a 24 h pre-culture process at 37 °C with 5% CO_2_. Once the cells fully adhered, the original culture medium was removed by aspiration. Cells in experimental groups were cultivated in medium supplemented with drugs, whereas blank control groups received medium without drug components. Following further incubation for 24 h and 48 h under 37 °C, 5% CO_2_, 10 μL CCK-8 solution was supplemented to every well and maintained for 1–4 h. Optical density values at 450 nm were detected via a microplate reader (Synergy H1, BioTek Instruments, Winooski, VT, USA).

### 4.8. Establishment of RAW264.7 Cell Inflammation Model

RAW264.7 cells were adjusted to 2 × 10^5^ cells/mL, and 100 μL of cell suspension was added to each well of the 96-well plates. After cell adhesion, the medium was discarded and grouped for modeling and drug treatment. Six parallel wells were prepared for each of the following experimental groups: the blank group was treated with 100 μL serum-free medium supplemented with 0.1% DMSO, the model group was treated with 100 μL LPS solution (1 μg/mL), the GA group was cultured in 100 μL serum-free medium mixed with GA stock solution, the BBR group was cultured in 100 μL serum-free medium mixed with BBR stock solution, the GB self-assembled hydrogel group was incubated with 100 μL serum-free medium loaded with GA/BBR self-assembled hydrogel, and the AMP antibiotic group received 100 μL AMP-containing serum-free medium. The final working concentrations in the drug-treatment groups were as follows: GA group, 20 μM GA; BBR group, 6.67 μM BBR; GB group, 20 μM GA + 6.67 μM BBR (molar ratio 3:1, consistent with the optimal formulation); AMP group, 10 μM AMP. After 2 h, 1 μg/mL LPS was added to all groups except the blank control group, followed by cell incubation for 24 h in a 37 °C and 5% CO_2_ environment. Cellular supernatant liquid was harvested and preserved at −80 °C for subsequent detection. 

### 4.9. ELISA Detection of TNF-α and IL-6

The frozen supernatants of each group were used as test samples. Standard concentration was set as the horizontal coordinate, and the corresponding optical density was adopted as the vertical coordinate to generate standard curves. The expression levels of TNF-α and IL-6 were calculated according to the regression curves.

### 4.10. Cell Scratch Assay

HaCaT keratinocytes were cultured to a confluent monolayer and scratched. After scratching, cells were washed twice with PBS, and images captured along the scratch edge served as the 0 h record. The cells were then treated with GB self-assembled hydrogel (final concentration: 20 μM GA + 6.67 μM BBR), free GA (20 μM), free BBR (6.67 μM), or serum-free medium as the control, and further cultured for 24 and 48 h. Wound closure was imaged and recorded at each time point.

Wound healing rate was calculated as follows: Logarithmic-phase HaCaT cells were digested and adjusted to a fixed seeding density of 1 × 10^5^ cells/mL. Seeded into each 6-well culture plate was 2 mL of the cell suspension. When cell confluence reached 80%, the culture medium was switched to serum-free basal medium for an additional 48 h incubation. When cell confluence reached 80%, the medium was replaced with FBS-free basic medium and cultured for another 48 h to achieve full confluence. The cell monolayer was scratched uniformly using a 200 μL pipette tip, then rinsed twice with PBS buffer to remove detached cells. Images were captured immediately after scratching as the 0 h baseline, followed by drug treatment and further incubation for 24 h and 48 h. The scratch width was measured at each time point, and the wound healing rate was calculated using the following formula:

(2) $$\mathrm{Wound}\;\mathrm{scratch}\;\mathrm{healing}\;\mathrm{rate}\;(\%)=\frac{d_{0,\mathrm{treatment}}-d_{t,\mathrm{treatment}}}{d_{0,\mathrm{treatment}}}\times 100\%$$

where d_0,treatment_ and d_t,treatment_ represent the scratch width of the treatment group at 0 h and at time t, respectively.

### 4.11. Antibacterial Activity

*S. aureus* (glycerol stock) was thawed and inoculated into TSB, followed by activation at 37 °C and 200 rpm for 6–12 h. *E. coli* (lyophilized powder) was rehydrated with sterile saline. The two strains were streaked onto corresponding solid media (*S. aureus*: TSA; *E. coli*: LB) and cultivated at 37 °C for 18–24 h. Typical single colonies were picked and subcultured to ensure purity. Single colonies in the logarithmic growth phase were suspended in sterile saline and adjusted to OD_600_ = 0.5 (≈1 × 10^8^ colony-forming units/mL) as standardized bacterial suspension.

For bacterial growth curve measurement, the standardized bacterial suspension was diluted to OD_600_ ≈ 0.05 in TSB (for *S. aureus*) or LB medium (for *E. coli*), then mixed with an equal volume of sample solution (final total mass concentration: 0.1 mg/mL). The mixtures were incubated at 37 °C with shaking at 200 rpm, and the OD_600_ value was measured every 1 h for 8 h. Sterile medium was used as a blank control for baseline correction, and each group was set with 3 parallel replicates. The inhibition rate at 8 h was calculated as

(3) $$\mathrm{Inhibition}\;\mathrm{rate}\;(\%)=\left(1-\frac{OD_{\mathrm{sample}}-OD_{\mathrm{blank}}}{OD_{\mathrm{control}}-OD_{\mathrm{blank}}}\right)\times 100\%$$

where Inhibition rate (%) represents bacterial growth inhibition rate; OD_sample_ is the optical density of the sample-treated group; OD_blank_ is the optical density of the sterile medium blank for baseline correction; OD_control_ is the optical density of the bacteria-only positive control group.

The agar plate spreading count assay was performed under the same total mass concentration (0.1 mg/mL). To ensure the comparability of the experimental results, the concentration of single drug groups (GA, BBR) was set to be equal to the corresponding component content in the GB hydrogel group at the same total mass concentration, i.e., the GA and BBR contents in the 0.1 mg/mL GB group were consistent with the concentrations in the single GA and single BBR groups, respectively. This setting was adopted to evaluate whether the co-assembly system could achieve superior efficacy on the basis of equal active ingredient content.

Afterwards, each sample liquid was blended with 100 μL bacterial suspension calibrated to OD_600_ ≈ 0.1, followed by 24 h co-cultivation at 37 °C. A volume of 20 μL of mixed bacterial liquid was transferred onto solid agar plates and uniformly distributed using sterile glass beads. All culture dishes were placed upside down and incubated at 37 °C over 18–24 h. The number of colony-forming units was recorded to quantify the antibacterial capacity of the samples.

### 4.12. Hemolysis Assay

The 2% red blood cell (RBC) suspension was configured as follows. Briefly, 5 mL of whole blood was blended with 0.2 mL of anticoagulant and centrifuged at 2000 rpm for 5 min, followed by the removal of the supernatant. The erythrocyte pellet was rinsed with ten-fold the volume of normal saline repeatedly until the supernatant appeared transparent and colorless. Afterwards, 0.9% NaCl aqueous solution was adopted to dilute the washed RBCs to obtain a 2% suspension. Lyophilized powder of the GB hydrogel was dissolved in PBS buffer to generate serial concentrations: 2.00, 1.00, 0.50, 0.25, and 0.10 mg/mL. Every prepared solution was blended with RBC suspension at a 1:1 volume proportion, maintaining the final RBC concentration at 2%. After 4 h incubation at 37 °C, all mixtures were centrifuged for 5 min at 3500 rpm. A microplate reader was applied to record supernatant absorbance (A) at a wavelength of 540 nm. Triplicate parallel samples were arranged for each concentration group. PBS buffer served as the negative control, while distilled water was set as the positive control group. The hemolysis percentage was computed via the formula listed below:

(4) $$\mathrm{Hemolysis}\;\mathrm{rate}\;(\%)=\frac{A_{\mathrm{sample}}-A_{\mathrm{negative}}}{A_{\mathrm{positive}}-A_{\mathrm{negative}}}\times 100\%\;$$

where A_sample_, A_positive_, and A_negative_ are the absorbance readings of the sample, positive control, and negative control, respectively.

### 4.13. Establishment of Diabetic Model

#### 4.13.1. Preparation of STZ Solution

Solution A was prepared by dissolving 2.10 g of citric acid in 100 mL of double-distilled water, while solution B was obtained by dissolving 2.94 g of sodium citrate in an equal volume of double-distilled water. Citrate buffer was subsequently formulated by blending solution A and solution B at a volume ratio of 1:1, followed by pH adjustment to the range of 4.2–4.4. Afterwards, 1.0 g of STZ was dissolved in the above-prepared citrate buffer and fixed to a final volume of 100 mL. The prepared solution was kept on ice and injected within 30 min.

#### 4.13.2. Model Establishment and Grouping

All experimental mice were assigned randomly to five treatment cohorts, namely the healthy group, blank group, AMP group, GB group, and commercial group, containing twelve individuals in each cohort. Prior to STZ injection, the mice were subjected to strict 12 h overnight fasting (with unrestricted water intake) to empty the intestinal contents and stabilize baseline blood glucose levels. Subsequently, with the exception of the healthy control group, the remaining blank, AMP, GB, and commercial groups were administered a single intraperitoneal injection of 1% STZ citrate buffer solution at a uniform dose of 200 mg/kg body weight to induce type 1 diabetes. To prevent death from hypoglycemia caused by excessive insulin secretion, standard rodent chow and drinking water containing 20% sucrose were provided continuously for 24 h following injection; thereafter, the sucrose-containing water was replaced with pure distilled water for routine feeding. Within 24 h of STZ injection, tail vein blood glucose levels were measured using a portable glucose meter; then, on days 3, 5, and 7 post-injection, fasting blood glucose was measured again after a 4–6 h fast. Mice exhibiting fasting blood glucose levels consistently ≥16.7 mmol/L for 7 consecutive days, accompanied by typical diabetic symptoms (polydipsia, polyphagia, and polyuria), were defined as stable type 1 diabetes models. Individuals exhibiting transient hyperglycemia or blood glucose levels exceeding 33.3 mmol/L were culled and excluded from subsequent wound surgery. Only mice confirmed to maintain sustained, stable hyperglycemia were selected to establish a full-thickness infected wound model, as persistent, long-term hyperglycemia mimics the pathological microenvironment of clinical diabetic foot ulcers—characterized by persistent inflammation and impaired angiogenesis—making this model suitable for evaluating the therapeutic effect of hydrogel on diabetic chronic wound repair.

### 4.14. Diabetic Wound Healing Rate Measurement

A full-thickness skin defect model was constructed on the back of diabetic mice under sterile conditions. Diabetic mice received intraperitoneal anesthesia via 1% sodium pentobarbital solution at a dosage of 100 mg/kg, followed by depilation of the dorsal skin. Circular full-thickness cutaneous wounds with a diameter of 1 cm were established on the dorsal skin of mice using a biopsy punch. Mixed bacterial suspension was seeded over the wound surface to set up the infected wound animal model. The mixed bacterial suspension contained equal volumes of *E. coli* and *S. aureus*, each adjusted to a concentration of 1 × 10^7^ CFU/mL. A total volume of 20 μL of the mixed suspension was evenly inoculated onto the wound surface. After 30 min of bacterial adsorption, the corresponding dressings were applied to each group to start the treatment. For the AMP group, ampicillin solution was prepared with sterile saline, and 20 μL of 1 mg/mL AMP solution was applied to each wound, with the dose equivalent to 20 μg per wound. The commercial hydrogel dressing used in this study was a sodium alginate-based medical hydrogel wound dressing (Wego Yourui, Weihai, China), which is clinically used for moist wound care and exudate management. The same mass (3.47 mg) of commercial dressing was applied per wound as the GB hydrogel group. After successful modeling, each mouse was treated with a fixed dose of 3.47 mg hydrogel dressing applied evenly to the wound area, and the wound was covered and fixed with medical polyurethane transparent film. The dressings were replaced every two days throughout the experimental period.

Wound images were taken on days 3, 7, and 14 post-surgery, and the wound closure area was quantitatively analyzed using ImageJ 1.53t software. On days 7 and 14, the mice were euthanized, and tissue blocks containing the wound center and adjacent normal skin were completely excised, fixed properly, and subjected to histopathological analysis.

### 4.15. In Vivo Anti-Inflammatory Effect by ELISA

Wound tissues of the same size were collected from each group, minced in normal saline, and centrifuged at 3000 rpm for 10 min, and the supernatant was stockpiled at −80 °C. The levels of TNF-α and IL-6 were measured according to the ELISA kit instructions.

### 4.16. Wound Healing Evaluation by H&E Staining

Samples were prepared according to established protocols [40]. Subsequently, micrographs of wound tissue sections were captured, and ImageJ 1.53t software was applied for quantitative detection of the thickness of regenerated epidermis and granulation tissue.

### 4.17. Masson Staining Evaluation

Optical microscopic observation was performed on wound tissue slices stained with Masson reagent, followed by micrograph collection. The relative collagen content in wounds at each time point was quantitatively analyzed using ImageJ software.

### 4.18. Angiogenesis Evaluation by Immunofluorescence Double Staining

CD31 and α-SMA immunofluorescence double staining was performed to evaluate neovascularization in wounds. Images were captured under a fluorescence microscope (BX53, Olympus, Tokyo, Japan), and the positive area ratios of CD31 and α-SMA were statistically analyzed by threshold segmentation using ImageJ 1.53t to quantify angiogenesis.

### 4.19. Statistical Analysis

All experiments were performed with at least three independent replicates, and the data are presented as mean ± standard deviation (SD). Statistical analysis was conducted using GraphPad Prism 9 software. For experiments with single-factor grouping (e.g., cytotoxicity, antibacterial rate at single time point), the differences between multiple groups were evaluated by one-way analysis of variance (ANOVA) followed by Tukey’s post hoc test. For outcomes involving both treatment and time factors (e.g., wound healing rate, tissue staining quantitative results at multiple time points), two-way ANOVA followed by Tukey’s post hoc test was used for statistical analysis. For histological and immunofluorescence quantification, 6 mice were analyzed per group at each time point, 3 tissue sections were prepared per sample, and 5 random microscopic fields per section were selected for quantitative analysis. All outcome assessments were performed by two independent researchers in a blinded manner to avoid subjective bias. The statistically significant differences are expressed as * *p* < 0.05, ** *p* < 0.01, *** *p* < 0.001 compared with the control group.

## Data Availability

The data presented in this study are openly available in the article.

## Supplementary Materials

The following supporting information can be downloaded at: https://www.mdpi.com/article/10.3390/gels12090799/s1, Figure S1: Specificity test of the reference standards and test samples (a: BBR; b: GA); Figure S2: Standard curves (a: BBR; b: GA); Table S1: Results of the GA and BBR precision test (n = 6); Table S2: Stability test results (n = 6); Table S3: Reproducibility test results (n = 6); Table S4: Spike recovery results of BBR and GA (n = 6).
