# Supplementary material for: Study on Berberine/Glycyrrhizic Acid Monoammonium Salt Self-Assembled Hydrogel for Diabetic Wound Healing
Source: Gels. 2026 Sep 2;12(9):799. doi: 10.3390/gels12090799 (PMC13606301; doi:10.3390/gels12090799)
Supplement: Supplementary file 1 [file gels-12-00799-s001.zip › gels-4449298-supplementary.pdf]

1 Experimental Methods

1.1 Establishment of HPLC analytical method for GB self-assembled hydrogel

1.1.1Chromatographic conditions

A Diamonsil C18 column (4.6 × 250 mm, 5 μm) was adopted. The mobile phase consisted of acetonitrile (A)0.1% aqueous phosphoric acid solution (B). Gradient elution was performed (0–20 min, 30%–65% A). The flow rate was set to 1.0 mL/min, the injection volume was 10 μL, column temperature was maintained at 25 °C, and the detection wavelength was 280 nm.

1.1.2 Preparation of reference standard solutions

Accurately weighed 15.00 mg of GA reference standard and 15.10 mg of BBR reference standard were transferred into a 10 mL volumetric flask. Methanol was added to the mark. The mixture was sonicated for 10 min, cooled to ambient temperature, and methanol was supplemented to the scale mark. The solution was filtered through a 0.22 μm microporous membrane to obtain the mixed standard stock solution.

1.1.3 Preparation of test sample solutions

Approximately 5 mg of lyophilized GB self-assembled hydrogel powder was accurately weighed and transferred into a 50 mL volumetric flask. Methanol was added to the mark. The solution was sonicated for 30 min, cooled to room temperature, and methanol was replenished to the scale mark. The resulting solution was filtered via a 0.22 μm microporous membrane to prepare the test sample solution.

1.1.4 Specificity

Aliquots (10 μL) of the above-prepared test sample solution and mixed reference standard solution were injected separately under the chromatographic conditions described in Section 1.1.1.

1.1.5 Linearity

Appropriate volumes of mixed standard stock solution were diluted with methanol to obtain a series of working standard solutions. The concentration series for GA were 2.34, 4.69, 9.38, 18.75, 37.50, 75.00, and 150.00 μg/mL; for BBR, concentrations were 2.36, 4.72, 9.44, 18.88, 37.75, 75.50, and 151.00 μg/mL. Each solution was determined according to the chromatographic conditions in Section 1.1.1. Calibration curves were plotted with concentration (x) as the abscissa and peak area (y) as the ordinate.

1.1.6 Precision

The mixed reference standard solution was filtered through a 0.22 μm microporous membrane

with an injection volume of 10 μL. Intra-day precision was assessed by six consecutive injections within one single day. Inter-day precision was evaluated by performing six injections per day for three consecutive days. Peak areas of GA and BBR were recorded, and relative standard deviation (RSD) values were calculated.

1.1.7 Solution stability

The test sample solution prepared in Section 1.1.3 was filtered with a 0.22 μm microporous membrane. Aliquots were injected at 0, 2, 4, 8, 10, and 12 h (injection volume = 10 μL). Peak areas of GA and BBR were recorded for calculation of RSD values.

1.1.8 Repeatability

Approximately 5 mg aliquots of lyophilized GB hydrogel powder were accurately weighed (six parallel samples). Test sample solutions were prepared following Section 1.1.3 and analyzed under chromatographic conditions described in Section 1.1.1. Peak areas and contents of GA and BBR were determined, and corresponding RSD values were calculated.

1.1.9 Spike-recovery test

Six portions of GB hydrogel test samples with known analyte contents were accurately weighed, and corresponding reference standard substances were spiked into each sample. Sample solutions were prepared as described in Section 1.1.3 and analyzed under chromatographic conditions of Section 1.1.1. The recoveries and RSD values were calculated.

2 Results and Discussion

2.1 Specificity

The reference standard solutions and test sample solution were subjected to HPLC measurement, as shown in Figure S1. The chromatograms exhibited well-defined peak shapes, satisfactory response intensity and favorable peak separation, demonstrating acceptable specificity of this analytical method.

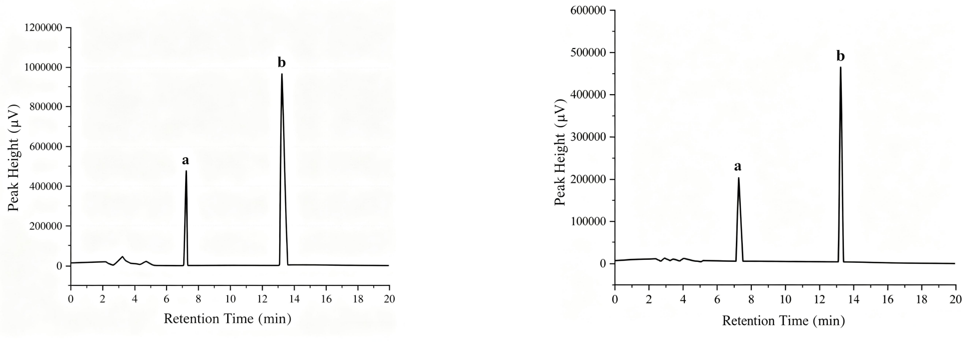

Figure S1. Specificity test of the reference standards and test samples (a: BBR; b: GA)

2.2 Linearity

Calibration curves were constructed using concentration as x-axis and peak area as y-axis. The regression equations were y = 29812x − 26202 (R^2 = 0.9999) for BBR and y = 14204x − 49387 (R^2 = 0.9994) for GA. Good linear relationships were observed for GA within 2.34–150.00 μg/mL and for BBR within 2.36–151.00 μg/mL, satisfying the requirements for method validation.

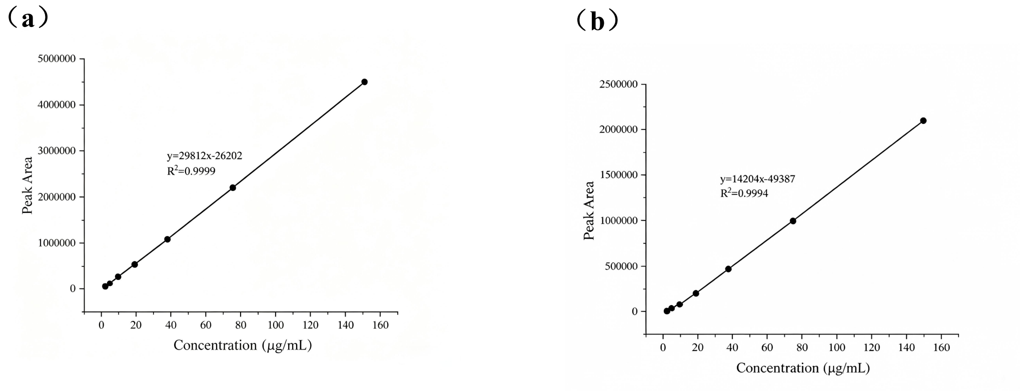

Figure S2. Standard curves (a: BBR; b: GA)

2.3 Precision test

Peak-area data for GA and BBR (n = 6) are summarized in Table S1. All RSD values were less than 2%, indicating acceptable precision of this quantification method.

Table S1 Results of the GA and BBR precision test (n = 6)

Sample	BBR peak area	GA peak area
1	804379	1582860
2	798907	1587691
3	790763	1590032
4	813009	1585141
5	784563	1576091
6	800461	1587023
Mean	795347	1584806
RSD	0.86 %	0.314 %

2.4 Stability test

Stability test results are presented in Table S2. All RSD values were below 2%, proving that the prepared sample solution remained stable within 12 h.

Table S2 Stability test results (n = 6)

Sample	BBR peak area	GA peak area
1	494379	808162
2	489987	816890
3	492371	810019
4	491175	807901
5	490097	809901
6	491067	810752
Mean	491512	810604
RSD	0.34 %	0.40 %

2.5 Repeatability test

The repeatability data are listed in Table S3. All RSD values were less than 2%, which confirmed good repeatability for the developed method.

Table S3 Reproducibility test results (n = 6)

Sample	BBR peak area	GA peak area
1	482681	819899
2	481198	800116
3	490036	818879
4	485759	809076
5	486780	811123
6	490651	811567
Mean	481684	811776
RSD	0.78 %	0.89 %

2.6 Spike-recovery test

The spike-recovery outcomes are shown in Table S4. Recoveries for GA and BBR were both greater than 90%, with all RSD values less than 2%. The results met the acceptance criteria for analytical method validation.

Table S4. Spike recovery results of BBR and GA (n = 6)

Sample	No.	Known content of sample	Added reference standard	Total measured content (μg)	Recovery (%)	Mean recovery (%)	RSD （%）

		(μg)	(μg)				
	1	17.46	17.50	34.99	100.09		
	2	17.46	17.50	35.03	100.20		
BBR	3	17.46	17.50	34.72	99.31	99.87	0.38
	4	17.46	17.50	34.80	99.54		
	5	17.46	17.50	34.90	99.83		
	6	17.46	17.50	35.05	100.26		
	1	32.40	32.50	64.85	99.92		
	2	32.40	32.50	64.88	99.97		
GA	3	32.40	32.50	64.73	99.74	99.92	0.25
	4	32.40	32.50	65.06	100.25		
	5	32.40	32.50	64.61	99.55		
	6	32.40	32.50	64.94	100.06		
